# Influence of Trp-Cage on the Function and Stability of GLP-1R Agonist Exenatide Derivatives

**DOI:** 10.1021/acs.jmedchem.4c01553

**Published:** 2024-09-10

**Authors:** Dániel Horváth, Pál Stráner, Nóra Taricska, Zsolt Fazekas, Dóra K. Menyhárd, András Perczel

**Affiliations:** † Medicinal Chemistry Research Group, HUN-REN Research Centre for Natural Sciences, Magyar Tudósok Körútja 2, H-1117Budapest, Hungary; ‡ HUN-REN−ELTE Protein Modeling Research Group, ELTE Eötvös Loránd University, Pázmány Péter sétány 1/A, Budapest H-1117, Hungary; § Laboratory of Structural Chemistry and Biology, ELTE Eötvös Loránd University, Pázmány Péter sétány 1/A, Budapest H-1117, Hungary; ∥ Hevesy György PhD School of Chemistry, ELTE Eötvös Loránd University, Pázmány Péter sétány 1/A, Budapest H-1117, Hungary

## Abstract

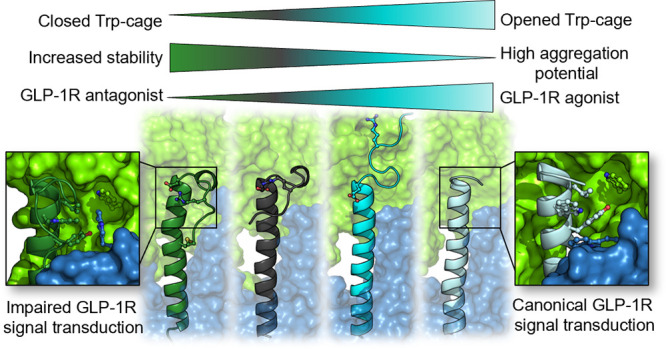

Exenatide (Ex4), a GLP-1 incretin mimetic polypeptide, is an effective therapeutic agent against diabetes and obesity. We highlight the indirect role of Ex4’s structure-stabilizing Trp-cage (Tc) motif in governing GLP-1 receptor (GLP-1R) signal transduction. We use various Ex4 derivatives to explore how Tc compactness influences thermal stability, aggregation, enhancement of insulin secretion, and GLP-1R binding. We found that Ex4 variants decorated with fortified Tc motifs exhibit increased resistance to unfolding and aggregation but show an inverse relationship between the bioactivity and stability. Molecular dynamics simulations coupled with a rigid-body segmentation protocol to analyze dynamic interconnectedness revealed that the constrained Tc motifs remain intact within the receptor–ligand complexes but interfere with one of the major stabilizing contacts and recognition loci on the extracellular side of GLP-1R, dislodging the N-terminal activating region of the hormone mimetics, and restrict the free movement of TM6, the main signal transduction device of GLP-1R.

## Introduction

Type 2 diabetes mellitus (T2DM) is one of the fastest growing global health emergencies of the 21st century, affecting more than half a billion people. This condition places an enormous burden on public healthcare systems worldwide, with the coupled annual healthcare expenditures approaching one trillion USD.^1^ The pathophysiological background of T2DM is not fully understood, but it is most commonly associated with overweight and obesity-related insulin resistance, which goes hand-in-hand with an increased risk of obesity-related cardiovascular diseases. Drugs that mimic the incretin effect^2^ of the gut-derived hormone glucagon-like peptide-1 (GLP-1), have been used in the treatment of T2DM since 2005, exploiting its glucose-dependent insulin secretion enhancing effect.^3^ Recent clinical trials have shown that GLP-1 receptor (GLP-1R) agonists significantly induce weight loss,^4,5^ reduce symptoms of heart failure, and improve the chances of a positive outcome in heart attacks and strokes.^6,7^ Given their diverse pharmacological profile,^8−12^ which allows for the simultaneous treatment of various obesity-related diseases, it is not surprising that GLP-1-based drugs were selected as “Breakthrough of the Year medications” in 2023 by Science.^13^

**Figure 1:**
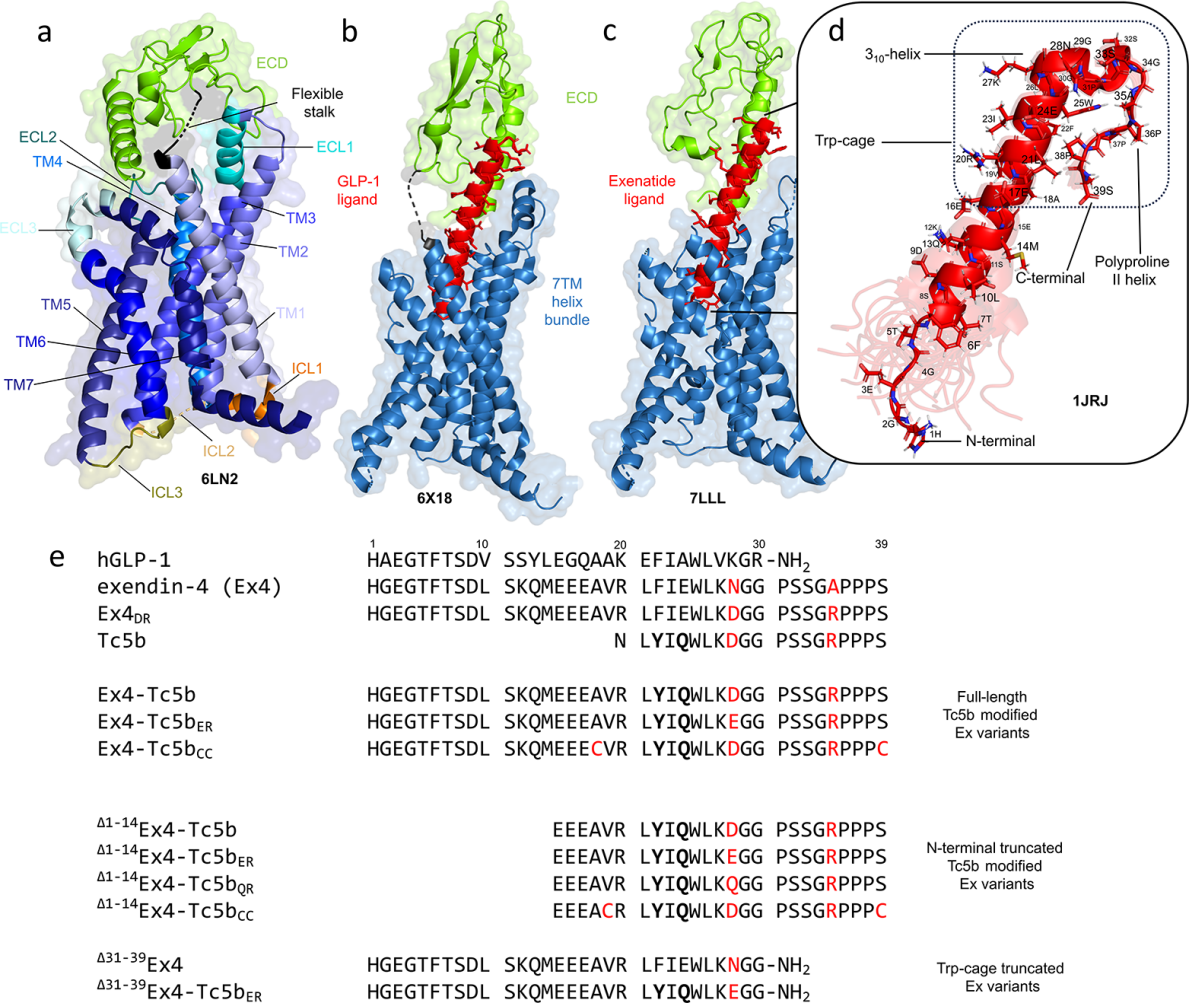
Structural overview of the inactive and active states of the GLP-1 receptor and the list of the Tc5b-modified exenatide sequences used in this study. (a) Crystal structure of the ligand-free and thus inactive state of the GLP-1R, including the 7TM region and the ECD domain. The extracellular domain (ECD, green), the transmembrane helices (TM1–7, shades of blue), the intracellular (ICL1–3, shades of orange), and the extracellular (ECL1–3, shades of turquoise) segments are highlighted. Cryo-EM-derived structures of the active states of GLP-1R with its orthosteric ligands (red) (b) GLP-1 and (c) exendin-4/exenatide (Ex4). Their flexible stalk (depicted in black) and some ICL and ECL were not built into the models (due to the absence of corresponding densities), indicating the flexibility of these regions even in the activated state. Interaction partners on the cytoplasmic side (Ras-like domain of Gαs in complex with Gβ, Gγ, and Nb35) docked to the activated TM domain are not shown. (d) NMR ensemble of structures of Ex4 determined in 30% v/v trifluoroethanol-containing water. Ex4 adopts a Trp-cage tertiary structure, formed by the C-proximal helical part, a 3_10_ helix, and a polyproline II helix, encompassing the central W25 residue. The α-helical part is also recognized by the ECD domain, whereas the latter two segments are absent in the cryo-EM structure of GLP-1R-bound Ex4 (c). In contrast, the N-terminal part of the ligand is highly dynamic in the solution (d) but becomes well-defined within the 7TM binding pocket of the receptor–ligand complex (c). (e) Single-letter amino acid sequences of Tc5b-modified Ex4 derivatives in this study. The superscript on the left indicates the number of residues of the truncated variants, while the subscript on the right indicates the applied amino acid mutation compared to the Ex4-Tc5b base sequence. Amino acid residues within the sequence at positions highlighted in red may contribute to either salt bridge formation or disulfide cross-linking.

GLP-1R belongs to the class B1 GPCR family.^14^ GLP-1R shares the canonical GPCR architecture of the seven-transmembrane structure (7TM), formed by the predominantly helical transmembrane domains (TM1–TM7) connected by three extracellular (ECL1–ECL3) and three intracellular loops (ICL1–ICL3).^15,16^ Ligand recognition is assisted by a specific extracellular N-terminal domain^17−19^ (ECD), which is attached to the 7TM by a flexible linker sequence, the stalk (Figure 1a). While the architecture of the 7TM is highly similar across classes, the ECL and ECD regions show considerable structural diversity, allowing each class to interact with a wide range of ligands that differ in size and physicochemical nature.^20^ The binding mode of polypeptide ligands to GLP-1R is also canonical. The C-terminal part of the ligand is essential for the recognition of the ECD domain of the GPCR, while the N-terminal region of the peptide interacts with the binding cavity of the 7TM region, promoting a structural rearrangement, which in turn induces an activation signal (Figure 1b,c) referred to as the two-domain binding mode. Truncation of the N-terminal part of the ligand polypeptide results in the loss of biological activity, while the capacity to interact with the ECD remains, resulting in an antagonistic effect.^21,22^ A prerequisite for ECD preselection seems to be the α-helix-forming potential of the ligand.^15^

Exendin-4 (Ex4), also known by its generic trade name exenatide, a 39-amino acid-long polypeptide isolated from the saliva of the Gila monster lizard,^23^ is a potent GLP-1R agonist.^26^ It shares a 53% sequence similarity in its first 30 amino acids with human GLP-1 and adopts a nascent helical structure that allows it to effectively bind to GLP-1R^23,24^ (Figure 1c,d). In addition, Ex4 features a proline-rich C-terminal extension of 9 residues, arranged as a short 3_10_ helix followed by a polyproline-II helical tail. These structural components flank an evolutionary conserved Trp residue, W25, forming a so-called Trp-cage (Tc) motif.^25^ Runge has shown that Ex4 has a higher affinity for the isolated ECD of the GLP-1R than GLP-1 does, while the Tc itself does not influence receptor interactions or contribute to the enhanced α-helical propensity of Ex4.^26^ The sequence differences between GLP-1 and Ex4 are reflected in their distinct pharmacokinetic and pharmacodynamic profiles. To date, a vast arsenal of Tc-containing hormone mimetics has been developed and used in treatment,^27^ but the comprehensive analysis of these variants is beyond the scope of this work. Here, we focus on investigating how the Tc motif affects the structure and stability of the ligand and its insulinotropic potential. The Ex4 sequence was pivotal in creating the Tc5b polypeptide^28,29^ (Figure 1e), a pioneering, rationally designed 20-residue Tc model miniprotein, that has subsequently influenced several de novo design approaches.^30,31^ Due to its compact and ordered structure, Tc5b has also become a testing ground for both experimental and computational studies aimed at understanding the fundamental processes of protein folding and unfolding.^32,33^

In addition to the various hydrophobic interactions that predominantly stabilize the Tc, a fold-stabilizing salt bridge has been introduced by the N28D and G35R substitutions (residue numbering is according to the original Ex4 scheme, Figure 1e). At neutral pH, this salt bridge contributes ∼4–5 kJ/mol to the overall stability of the fold, which is slightly less than expected,^29^ suggesting a less than ideal orientation of the participating residues. The search for the optimal side chain combination to participate in such a salt bridge has yielded inconclusive results, and the observed differences remain unexplained at the atomic level.^34,35^ Alternative strategies were explored to further improve both the folded population ratio and the thermal stability of the Tc fold. One such approach involves the introduction of two cysteines (A18C and S39C),^36^ whose close proximity leads to spontaneous oxidation and stable disulfide bond formation, effectively locking the Tc fold in place without affecting its binding mode to the isolated ECD of GLP-1R.^37^

GLP-1 derivatives, along with other B-class GPCR peptide ligands, exhibit an intrinsic propensity for amyloid aggregation,^38^ which is not entirely surprising since these hormone peptides are stored in acidic secretory vesicles inside the cell as functional amyloid fibrils before being released into the circulation. This aggregation is fully reversible; the amyloid deposits disintegrate into the biologically active monomeric form when released into the near neutral pH of the circulation.^39^ The main aggregation core of these peptides (between residues 21 and 26) overlaps with their receptor-binding helical segments and is also an integral part of the Tc fold. The switch between amyloid and folded monomeric forms initiated by the pH shift is focused on the evolutionary conserved glutamic acid (E21), which becomes protonated (at pH ∼5.2) and triggers amyloid formation of this aggregation-prone region (APR).^40^ Thus, oligomerization processes of polypeptide-based medicines are generally sensitive to environmental conditions;^41^ so, these parameters must be carefully optimized and controlled during the lifetime of the drug, to ensure the quality, efficacy, and safety.^42^

Here, we present a quality by design^43^ study, aimed at systematically probing the influence of Tc compactness and rigidity on the biological function of incretin analogues. As a first step, we introduced the Tc5b-specific modifications to the Ex4 sequence, yielding an Ex4-Tc5b chimera. We then modified the salt bridge and finally constrained and rigidified the Tc fold by creating a bridging disulfide bond (Figure 1e). We performed a comprehensive analysis of the thermal stability of these constructs at physiologically relevant neutral pH along with the characterization of their aggregation potential and bioactivity. Based on the results, we suggest an indirect but critical role for the C-terminal Tc motif in activating GLP-1R.

## Results

### Spectroscopic Characterization of the Thermal Unfolding of the N-Terminally Truncated Constructs

**Figure 2:**
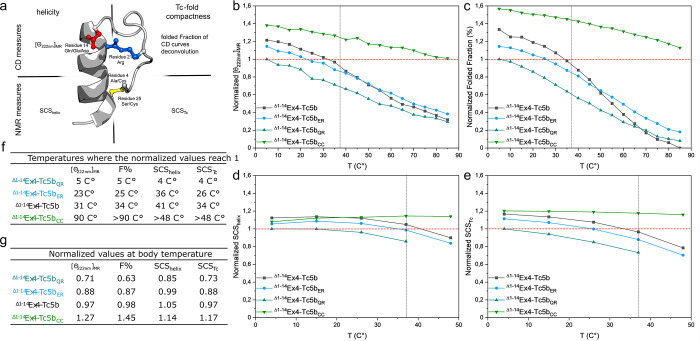
Comparison of measured melting curves using the normalized data sets at pH = 7. (a) Schematic overview of the applied biophysical approaches in characterization of the thermal unfolding of ^Δ1–14^Ex4-Tc5b variants. (b) Normalized molar ellipticity values at 222 nm [Θ_222nm_]_MR_ and (c) folded fraction values derived from deconvolution are obtained by far-UV CD spectroscopy. (d) Normalized sums of secondary chemical shift values describing helicity (SCS_helix_) and (e) Tc fold compactness (SCS_cage_) determined by NMR. In all four cases (b–e), larger values indicate a higher degree of structural order. Note that the NMR-derived data (4–48 °C) cover only a fraction of the temperature range used to record the CD spectra (5–85 °C). Each data set has been normalized to the respective data point corresponding to the ^Δ1–14^Ex4-Tc5b_QR_ measured at the lowest temperature. This standardizes the scale and units of the results obtained by different techniques, facilitating the comparison of the recorded melting curves. These reference data points are considered as 1. (f) Normalization of the measured data points allowed us to determine the temperatures at which the variants reach the same structural order as the reference ^Δ1–14^Ex4-Tc5b_QR_ at 4 °C (horizontal red dotted line, b–e). (g) Comparison of the fold descriptors at the highest temperature, where all four values could still be determined (vertical black line, b–e).

CD- and NMR-based techniques (Figure 2a) were applied to characterize the thermal unfolding of the Tc5b-modified exenatide constructs using N-terminally truncated variants (^Δ1–14^Ex4-Tc5b) (Figure 1e) at pH = 7. The discarded N-terminal segments of GLP-1 analogs are highly dynamic in the absence of the receptor and do not exert a fundamental impact on Tc fold compactness.^23,25,36^ Meanwhile, the absence of the spectral contribution of the flexible N-terminal segment facilitates the interpretation of the CD data and allows the application of nonisotope-labeled two-dimensional homonuclear ^1^H–^1^H NMR approaches for assignment and structure determination. ^Δ1–14^Ex4-Tc5b_ER_ was previously shown to adopt the properly folded Trp-cage (Tc) three-dimensional structure.^31^

Various methods can be applied to quantify the thermal stability of the Tc fold, including measurements of the compactness of the tertiary structure of the Tc and the degree of its α-helicity (Figure 2a). “Melting curves” for each variant were recorded in the temperature range of 5–85 °C by CD spectroscopy (Figure S1), and the collected spectra were deconvoluted into base components.^44^ A U-type base spectrum typical of an unfolded polypeptide chain and a C-type base spectrum characteristic of a 3_10_ or α-helix were obtained. The weight of the latter component is referred to as the folded fraction (*F*%/%, Figure S2a) and was used to quantify the global compactness of the Tc fold. The helical content of the Tc constructs was characterized by using the molar ellipticity values measured at λ = 222 nm (Θ_222nm_/deg cm^2^ dmol^–1^) (Figure S2b). In addition, we performed the complete backbone and side chain resonance assignment of the truncated variants, at five temperatures (4, 15, 26, 37, and 48 °C) using ^1^H NMR data. The sum of the Hα secondary chemical shift (SCS) values of selected backbone protons (residues 16–27) provides an independent measure of the helicity, SCS_helix_ (Figure S2c). In variants in which a well-ordered Tc is present, the indole side chain of the central Trp residue significantly influences the chemical shifts of selected neighboring residues. The sum of the SCS of the protons most affected by the aromatic ring current serves as an indicator of the compactness and degree of folding of the Tc motif (SCS_Tc_) (Figure 2b–e and Figure S2d).

Although the physical backgrounds of CD and NMR are different, the resulting melting curves exhibit similar tendencies (Figure 2b–e). When analyzing the 4 ≤ *T* ≤ 50 °C temperature range, all four measurements yield the same thermostability order: ^Δ1–14^Ex4-Tc5b_CC_ > ^Δ1–14^Ex4-Tc5b > ^Δ1–14^Ex4-Tc5b_ER_ > ^Δ1–14^Ex4-Tc5b_QR_. ^Δ1–14^Ex4-Tc5b_QR_, which resembles most closely the parent Ex4 sequence (because it cannot form the Tc-stabilizing salt bridge), exhibits the lowest thermostability. The NMR resonances of ^Δ1–14^Ex4-Tc5b_QR_ measured at *T* = 48 °C have broadened to such an extent that indicates significant loosening of the Tc. Replacement of Q28 (of ^Δ1–14^Ex4-Tc5b_QR_) with either E28 or D28 considerably increases the thermostability of the Tc fold, as suggested previously.^29,31,35^ In the presence of the introduced D28/E28-R35 salt bridges, the temperature at which these variants reach the reference fold compactness is increased by 20–30 °C (Figure 2f). ^Δ1–14^Ex4-Tc5b differs from ^Δ1–14^Ex4-Tc5b_ER_ only in a methylene group on the acidic Asp/Glu “pillar”. However, this small difference results in a considerable 5–8 °C increase in thermal stability favoring the Asp-Arg salt bridge. Interestingly, at *T* > 48 °C, the unfolding properties reverse: the residual Tc compactness of ^Δ1–14^Ex4-Tc5b at higher *T* becomes inferior to that of ^Δ1–14^Ex4-Tc5b_ER_. All applied methods unequivocally confirm the outstanding thermostability of ^Δ1–14^Ex4-Tc5b_CC_: this compact Tc fold remains stable even under extreme thermal conditions.

### Trp-Cage Thermal Stability Explained by the Differences of the NMR Ensembles

**Figure 3:**
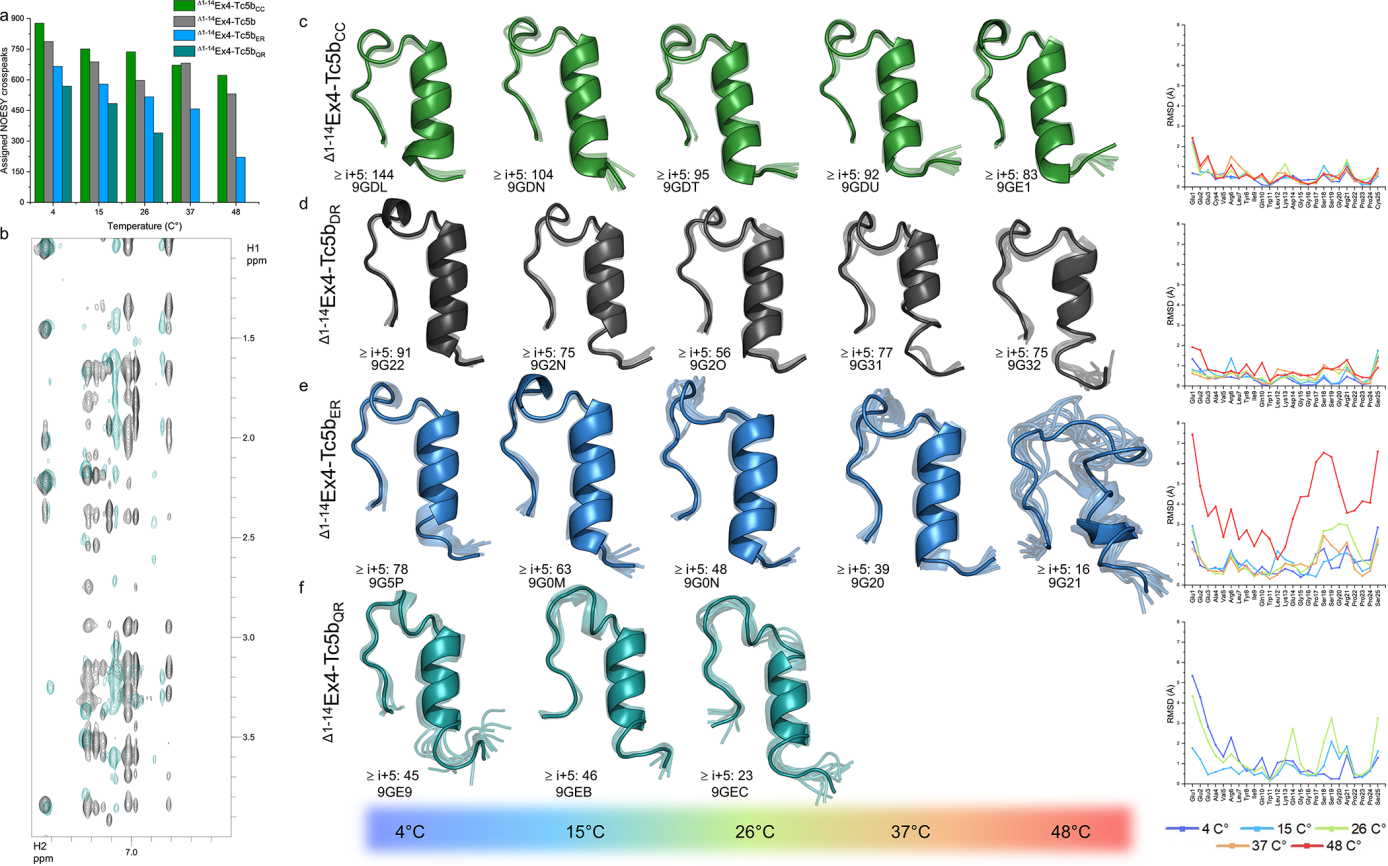
Analysis of the temperature-dependent Ex4-Tc5b chimera structural ensembles calculated using ^1^H–^1^H NOESY-derived constraints. (a) Number of assigned ^1^H–^1^H NOESY cross peaks at each temperature. (b) Aromatic region of the ^1^H–^1^H NOESY spectra of ^Δ1–14^Ex4-Tc5b (black) and ^Δ1–14^Ex4-Tc5b_QR_ (teal). Cross peaks corresponding to the side chains of Tyr8 and Trp11 were analyzed to determine the interaction network within the hydrophobic core of Tc. The abundance of the NOESY signals in this region provides valuable insight into the compactness of the Tc motif. An increase in the number of signals introduces additional restraints in the structural calculations, resulting in a more precisely defined and well-folded structure ensemble. (c–f) Structural ensembles (indicating their respective PDB entries) of Ex4-Tc5b derivatives as temperature elevates (left to right) as well as their all-atom RMSD values plotted along the sequence. Each ensemble consists of the 10 lowest energy structures calculated, shown in a semitransparent visualization, along with their average structure overlaid. Long-range NOESY cross peaks (≥*i* + 5), which are critical for determining the tertiary Tc motif, are shown adjacent to the structure ensembles.

To provide atomic-level insight into thermal unfolding, we performed NOESY constraint-based structure determination (Figure 3). A correlation was observed between the ranking of the Tc fold thermal stability (Figure 2b–e) and the number of assigned NOESY cross peaks (Figure 3a), especially with respect to long-range NOEs (Figure 3b–f). The number and distribution of the long-range NOEs are indicative of tertiary structure stability. At *T* > 26 °C, no inter-residue cross peaks could be assigned in the NOESY spectra of ^Δ1–14^Ex4-Tc5b_QR_, inferring the complete melting of the Tc fold. The extent of unfolding can be estimated and quantified by following the reduction in the number of cross peaks as a function of the increasing temperature. For example, at *T* = 48 °C, ^Δ1–14^Ex4-Tc5b_CC_ loses 29% of its total cross peaks; however, ^Δ1–14^Ex4-Tc5b loses 33%, while ^Δ1–14^Ex4-Tc5b_ER_ loses 67% (Figure 3a). The structures of ^Δ1–14^Ex4-Tc5b_ER_ at 48 °C are best described as a fuzzy and heterogeneous ensemble; however, the persistence of the remaining 16 long-range NOEs indicates the preservation of a cohesive core structure with topological similarity to the original Tc fold (Figure 3b). The slightly different ^Δ1–14^Ex4-Tc5b, which has Asp instead of Glu at position 28, retains its structural integrity even at 48 °C, according to the calculated ensemble, but CD measurements indicate that its Tc fold begins to dissolve just above 50 °C (Figures 2b,c and 3a).

The NMR ensembles with their respective RMSD values show that as the temperature increases, the Tc motifs gain a more and more molten globule character (Figure 3). All four variants share a similar temperature-driven unfolding mechanism; regardless of their sequential differences, the presence of the salt bridges simply delays the unfolding of the Tc motif (Figure 3c–f). The disulfide-cyclized ^Δ1–14^Ex4-Tc5b_CC_ shows extreme, toxin-like resistance to unfolding (Figure 3c). As the temperature increases, the loss of NOESY cross peaks indicates that two sites of the Tc fold experience more enhanced backbone dynamics. One of these is the N-terminus of the α-helix, and the other one is the 3_10_ helix of the −^29^GGPSSG^34^– segment, which first displaces and then becomes flexible. The effect of the increased internal motion extends toward the C-terminal part of the α-helix, destabilizing it from the opposite direction too. The polyproline helix remains tightly aligned and parallel oriented to the remaining helical core of the α-helix, suggesting that the mainly proline–aromatic interactions located here resist denaturation the longest. The final step is the collapse of the Tc fold, as exemplified by both ^Δ1–14^Ex4-Tc5b_QR_ and ^Δ1–14^Ex4-Tc5b_ER_ (Figure 3e,f).

**Figure 4:**
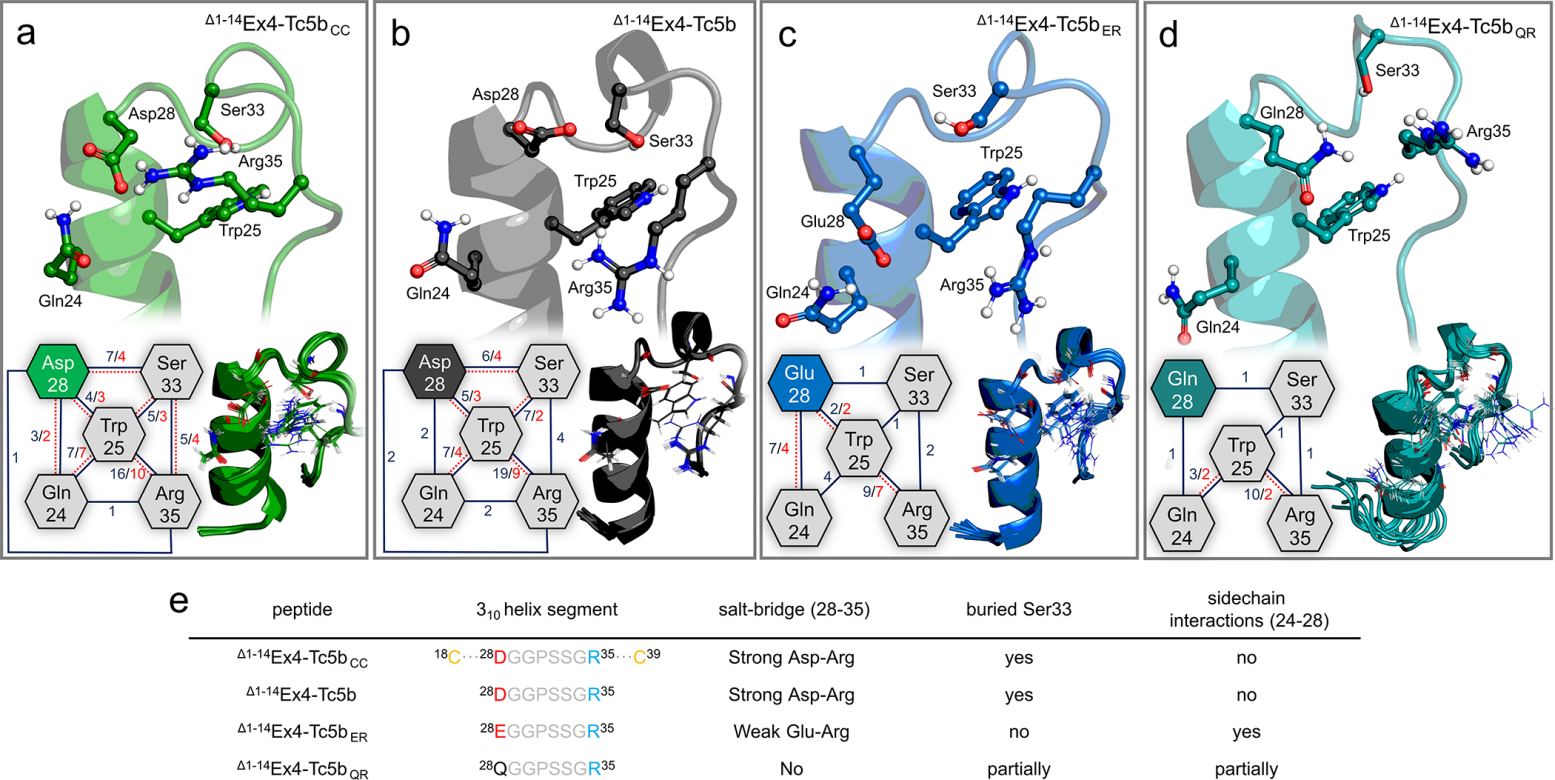
Comparison of the side chain interaction network modulating the 3_10_ helices. The molecular basis behind the observed thermostability differences of Tc variants lies in distinct interactions between residues Q24, W25, D/E/Q28, S33, and R35 (depicted in ball-and-stick representation) of the 3_10_ helix motifs for the following variants: (a) ^Δ1–14^Ex4-Tc5b_CC_, (b) ^Δ1–14^Ex4-Tc5b, (c) ^Δ1–14^Ex4-Tc5b_ER_, and (d) ^Δ1–14^Ex4-Tc5b_QR_. In the interaction networks (lower left corner), hexagons stand for the key residues, with connecting edges showing the number of the assigned NOE cross peaks at two temperatures: 4 °C (dark blue) and 26 °C (red). In water, the NMR-based assignment of some functional groups with exchanging protons, such as the guanidino group of Arg or the hydroxyl group of Ser, is challenging. Therefore, the detection of some of these signals is indeed informative and underlines the robustness of the Tc fold (for NOE data, see Table S1). The network of these side chain interactions defines the geometry of the 3_10_ helices, shown as the NMR ensembles depicted in the bottom right corner of each panel. The decrease in the “edge” numbers is an indication of the degree of resistance to thermal unfolding of the 3_10_ helices. The protons of the observed D28-R35 salt bridge and the OH proton of S33 are both detected in the case of ^Δ1–14^Ex4-Tc5b_CC_ and ^Δ1–14^Ex4-Tc5b, showing the extent of their burial within the hydrophobic core of the miniprotein. The mere presence of such groups in the NMR spectra provides valuable information about the 3D structure. Table (e) summarizes the different interactions that contribute to the 3_10_ helix and Tc fold compactness.

The results thus suggest that the overall thermal resistance of the Tc fold is significantly influenced by the position and geometry of the 3_10_ helix. The NMR ensembles have revealed a cooperative network of interacting side chains, those of Q24, W25, D28, S33, and R35 contributing to the cohesiveness of the 3_10_ helix (Figure 4). The cross peaks between the guanidino group of R35 and the Hβ protons of D28 confirm the presence of the salt bridge at 4 °C. In addition, R35 envelopes the indole ring of W25, hindering water influx into the hydrophobic core. Notably, ^Δ1–14^Ex4-Tc5b_ER_ and ^Δ1–14^Ex4-Tc5b_QR_ lack such peaks for E28/Q28 (Figure 4c,d), indicating that R35 is more fixed to W25 in constructs with D28 (Figure 4a,b). The ^Δ1–14^Ex4-Tc5b_QR_ variant loses all cross peaks already at *T* > 27 °C, suggesting the melting of the 3_10_ helix and subsequent unfolding of the Tc fold in the absence of the salt bridge. Analysis of the interaction network shows that the side chain of E28 prefers to form H-bond interactions with Q24 of the α-helix rather than participate in the 28–35 salt bridge interaction (Figure 4c) contributing only partially to the stabilization of the 3_10_ helix, explaining why ^Δ1–14^Ex4-Tc5b_ER_ is more thermolabile than ^Δ1–14^Ex4-Tc5b at lower temperatures. However, the more pronounced Q24–E28 interaction contributes to the stabilization of the helix of ^Δ1–14^Ex4-Tc5b_ER_ at higher temperatures to a greater extent than ^Δ1–14^Ex4-Tc5b, as indicated by the CD melting curves (Figure 2b,c). In addition, the side chain of S33 is found to be buried in the original Tc5b motif,^29^ a feature similarly observed in case of our ^Δ1–14^Ex4-Tc5b and ^Δ1–14^Ex4-Tc5b_CC_ peptides. The hydroxyl group of S33 actively participates in H-bonding interactions with W25 and R35, further solidifying the 3_10_ motif. However, in the case of ^Δ1–14^Ex4-Tc5b_ER_, possibly due to crowding caused by the longer aliphatic chain of E28, S33 faces the solvent and loses this stabilizing contribution (Figure 4c).

### Amyloid Aggregation Is Hindered by the Trp-Cage

**Figure 5:**
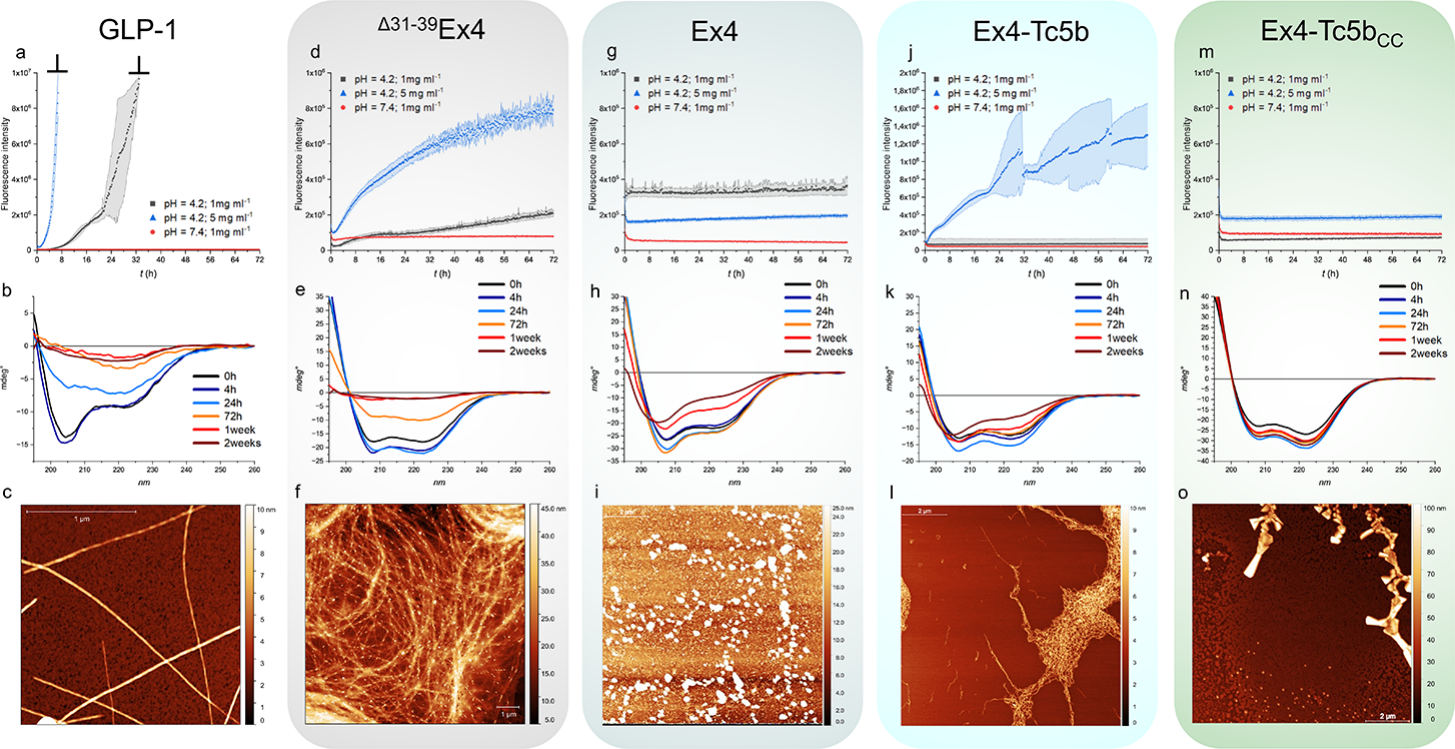
Amyloid aggregation propensity of GLP-1 and the related Ex4-Tc5b analogues. The aggregation behavior of GLP-1 (a–c), Tc-truncated Δ^31–39^Ex4-Tc5b (d–f), full-length Ex4 (g–i), Ex4-Tc5b (j–l), and Ex4-Tc5b_CC_ (m–o) was characterized by using the thioflavin-T binding assay, CD spectroscopy, and AFM techniques. The first row shows the emitted fluorescence of thioflavin-T assays at acidic and neutral pH, at concentrations of 1 and 5 mg mL^–1^ (acidic conditions) collected over a 3-day-long period. The average fluorescence and standard deviation of three parallel measurements were plotted as error-band functions. Fluorescence of GLP-1 exceeded the limit of detection range (indicated by ⊥ in panel a). Long-term aggregation was also monitored by CD spectroscopy under acidic (second row) and neutral conditions (Figure S4) at a concentration of 1 mg mL^-1^. The initial CD spectra exhibit characteristic CD properties of mainly α-helical folded structures. Over time, observable decreases in intensity associated with unfolding (b–k) occur, rather than the distinctive α-helical to β-sheet transition (Figure S4f). However, AFM micrographs (third row) of the 72 h agitated acidic ThT samples, at concentrations of 1 mg mL^–1^ (c,f,i,o) and 5 mg mL^–1^ (l) reveal fibril formation (c,f,l). Objects revealed by AFM of granulated or rectangular topology are NaCl salt crystals, crystallized during the vacuum drying process (i,o).

The pH-sensitive, reversible amyloid aggregation of GLP-1 and related gastrointestinal hormones is an inherent and functionally critical property. They are stored as condensed amyloids at low pH before being released as monomeric, folded hormones at neutral pH. At the same time, the exact evolutionary origin^45^ of Ex4 with its functional role as a salivary toxin in lizards remains unknown.^45,46^ The difference in secretion pathways is notable: GLP-1 is secreted into the bloodstream, while Ex4 is secreted into the alimentary canal. This difference may suggest different potentials for aggregation. In terms of structure, the polyproline tail of the Ex4 Tc fold effectively shields the −^21^LFIEWL^26^− aggregation-prone region^40^ (APR) of the facing α-helix, while the similar −^21^EFIAWL^26^− helical segment of GLP-1 remains unshielded. We therefore investigated the short- and long-term aggregation behavior of GLP-1, Ex4, and related Tc5b-modified variants using an amyloid-specific thioflavin-T fluorescence assay, CD spectroscopy, and atomic force microscopy (AFM) at 37 °C (Figure 5).

GLP-1 forms twisted amyloid fibrils with alternating topologies of 5–10 nm diameter under acidic conditions in a concentration-dependent manner within 1 day (Figure 5a–c). Interestingly, GLP-1 also forms amyloid assemblies under neutral pH conditions, albeit in two weeks, as indicated by an emerging B-type CD spectrum (Figure S4f,k). Ex4, with the Tc structural motif intact, did not exhibit any evidence of amyloid formation under the conditions applied (Figure 5g,h). However, its C-terminally truncated variant missing the Tc fold (^Δ31–39^Ex4), an α-helix of the same length as GLP-1, showed a positive, concentration-dependent ThT response under acidic conditions (Figure 5d–f). The fluorescence intensity of ^Δ31–39^Ex4 is 1 order of magnitude lower than that of GLP-1, but AFM confirms the presence of an extended fibril network after 72 h of gentle shaking. This shows that the helical segment of Ex4, in itself, is prone to amyloid formation, but the C-terminal proline-rich Tc extension can effectively block fibrillization. However, unlike GLP-1, ^Δ31–39^Ex4 does not form fibrils under neutral conditions even after two weeks (Figure S4g). Tc5b derivatives have previously been shown to be prone to aggregation under specific conditions,^47,48^ but our full-length Ex4-Tc5b variant showed only a limited aggregation propensity, with fibril formation occurring at high concentrations (Figure 5j–l), reaffirming the protective power of the Tc motif against aggregation. When comparing the aggregation potential of Ex4 and Ex4-Tc5b, it is important to note that these two systems not only differ in the absence (Ex4) or presence (Ex4-Tc5b) of the salt bridge-forming pair at positions 28 and 35 but also in their APR sequences: −^21^LFIEWL^26^− in Ex4 while −^21^LYIQWL^26^− in Ex4-Tc5b (Figure 1e). The E24Q substitution that was originally introduced into the Tc5b sequence as an α-helix stabilizer QxxxD^29,49^ side chain interaction fundamentally enhances the aggregation potential. This effect can be attributed to the replacement of the gatekeeper glutamic acid (which electrostatically hinders the self-assembly when carrying negative charge) with a nonionizable glutamine in the middle of the APR, making the Tc5b sequence more likely to aggregate despite the APR-shielding Tc fold. However, covalent locking of the Tc, as seen in Ex4-Tc5b_CC_, permanently covers the APR, diminishing the aggregation of this more potent segment, keeping the structure folded over two weeks, even under harsh conditions (Figure 5m–o).

### Ex4-Tc5b Chimera-Induced Insulin Secretion Is Inversely Proportional to the Compactness of Its Trp-Cage Fold

**Figure 6:**
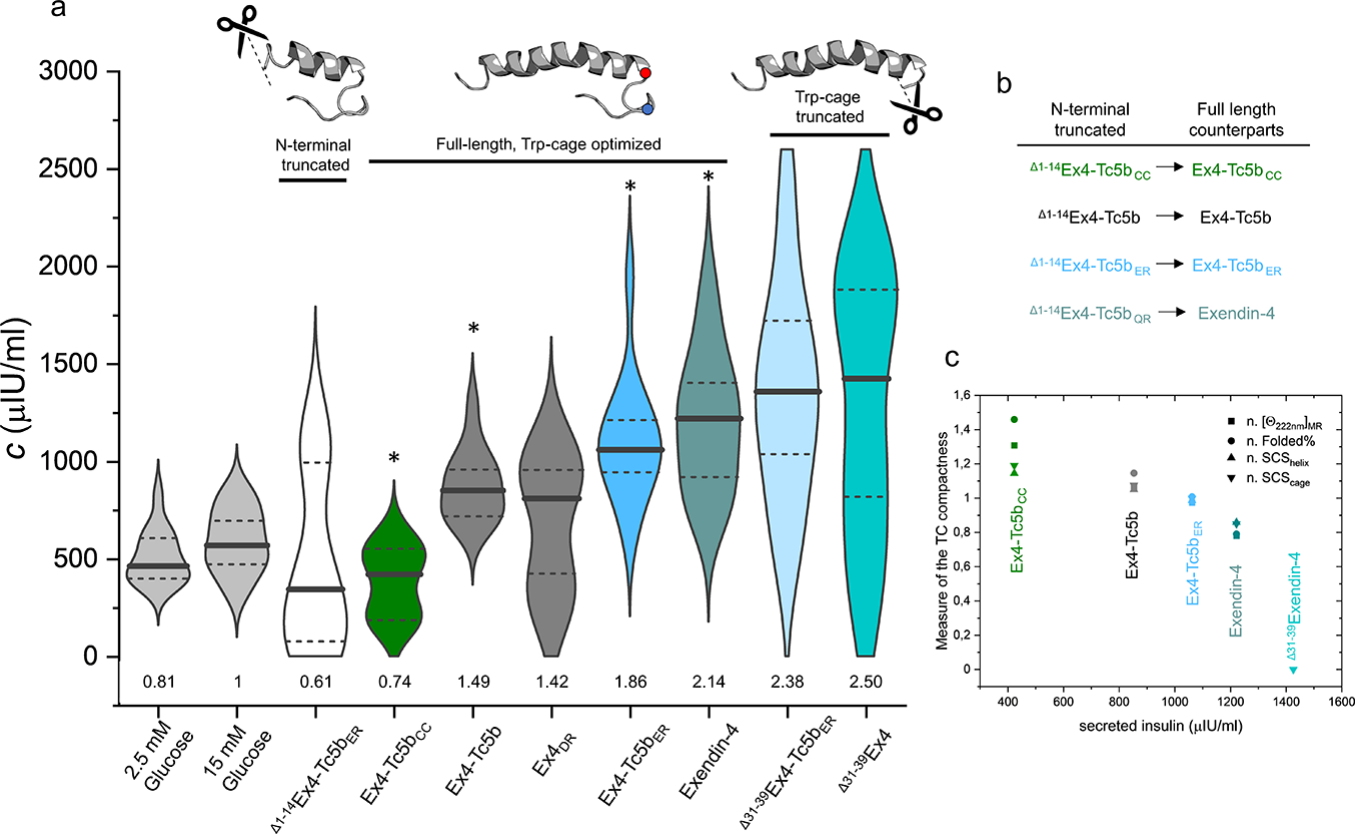
Insulin secretion enhancing potential of the Ex4-Tc5b variants in mammalian INS-1E cell cultures. (a) The violin plots illustrate the insulin secretion enhancing potential of Ex4-Tc5b variants in mammalian INS-1E cell cultures. Median values are represented by solid black lines, while the 25th and 75th percentile limits are represented by dashed black lines. A total of 14 absorbance measurements from 2-2 passed cell cultures per polypeptide were evaluated simultaneously. Glucose standards at low (2.5 mM) and high (15 mM) concentrations were used to confirm the adequacy of insulin secretion of the cell cultures. The cells were then treated with 15 mM glucose and 20 nM peptide. The values at the bottom of the violin charts indicate the fold increase in insulin secretion compared to the standard of 15 mM glucose. The receptor-activating segment truncated ^Δ1–14^Ex4-Tc5b_ER_ was used as a negative, while Ex4 was used as a positive control. Asterisks indicate the full-length variants whose truncated counterparts (see panel (b)) were tested for thermostability. It is noteworthy that despite the sequence differences, Ex4 was used as a full-length counterpart for ^Δ1–14^Ex4-Tc5b_QR_ since neither of these contain a Tc fold-stabilizing salt bridge. (c) The secreted insulin quantity in the presence of the full-length polypeptide is plotted against Trp-cage compactness, characterized by normalized (denoted as *n*) values at 25 °C of the truncated counterparts of the full-length peptides. The biological activity shows an inverse correlation with Tc fold compactness.

In addition to achieving improved thermostability and aggregation resistance, it is crucial to preserve the biological activity of the designed polypeptide hormones and drugs. To investigate the insulinotropic nature of the Tc fold derivatives, we measured the insulin secretion response to added glucose in rat INS-E cells in the presence of the different Ex4 and Ex4-Tc5b derivatives. The concentration of the secreted insulin was determined by enzyme-linked immunosorbent assays (Figure 6a). The ^Δ1–14^Ex4-Tc5b_ER_ peptide was used as a negative control, as it lacks the entire receptor-activating N-terminal segment, the bioactivity of the Trp-cage-optimized, full-length constructs (Ex4-Tc5b_CC_, Ex4-Tc5b_ER_, Ex4-Tc5b, and Ex4) was examined (Figures 1e and 6b). Since the N-terminal α-helical segment responsible for GLP-1R activation^31,36^ is identical across all of the full-length Ex4-Tc5b variants and they all contain the 3_10_ helix and polyproline tail of the Tc motif (Figure 3c–f), the observed differences in bioactivity can be attributed to sequence optimization of the Tc fold. In addition, two C-terminally truncated variants, ^Δ31–39^Ex4 and ^Δ31–39^Ex4-Tc5b_ER_, were tested where the shielding effect of the polyproline tail on the Trp core is absent, as in the case of human GLP-1.

The results of the insulin secretion assay and the Tc fold stability measurements (Figure 6c) showed an inverse correlation. The more compact and folded the Tc structure is, the less insulinotropic it is. In other words, receptor activation is more effective when the Tc cage motif is less stable and thus more prone to opening. Consistent with this, Ex4 variants lacking the Tc (^Δ31–39^Ex4 and ^Δ31–39^Ex4-Tc5b_ER_) show a superior biological potency, with a median 2.5-fold increase in insulin secretion as compared to that of 15 mM glucose, surpassing the potency of the Tc-containing variants. Among these, Ex4 outperforms all variants containing salt bridge-optimized (Ex4-Tc5b_ER_, Ex4_DR_, and Ex4-Tc5b) and disulfide-locked (Ex4-Tc5b_CC_) Tc motifs. In fact, Ex4-Tc5b_CC_, with a covalently locked Tc fold, induced no increase in secreted insulin, suggesting no receptor activation potential. Therefore, even though the C-terminal polyproline segment is not directly involved in binding to or activating the GLP-1R, its presence and conformational inadaptability exert a decisive influence on receptor activation. Structure comparison of the GLP-1R/ligand complexes available to date (Figure S5) shows that displacement of the polyproline tail upon binding to the ECD may be beneficial but is not a requirement. To gain a clearer understanding of the observed structure–activity relationship, we performed molecular dynamics simulations of the Ex4-Tc5b variants using the GLP-1R model.

### Molecular Dynamics (MD) Simulations of the Receptor–Ligand Complexes Reveal that the Fortified Tc Folds Stay Intact within the Complexes but Interfere with the ECL1 Receptor Domain

Simulations were performed to characterize the GLP-1R-bound ^Δ31–39^Ex4-Tc5b_ER_, Ex4-Tc5b_ER_, Ex4-Tc5b, and Ex4-Tc5b_CC_ complexes and to study the effect of the gradual tightening of the Tc fold, in comparison with the reference GLP-1/GLP-1R complex. A common model assembly was created as a starting structure for all the simulations, based on the cryo-EM structure of the GLP-1/GLP-1R complex (6X18).

**Figure 7:**
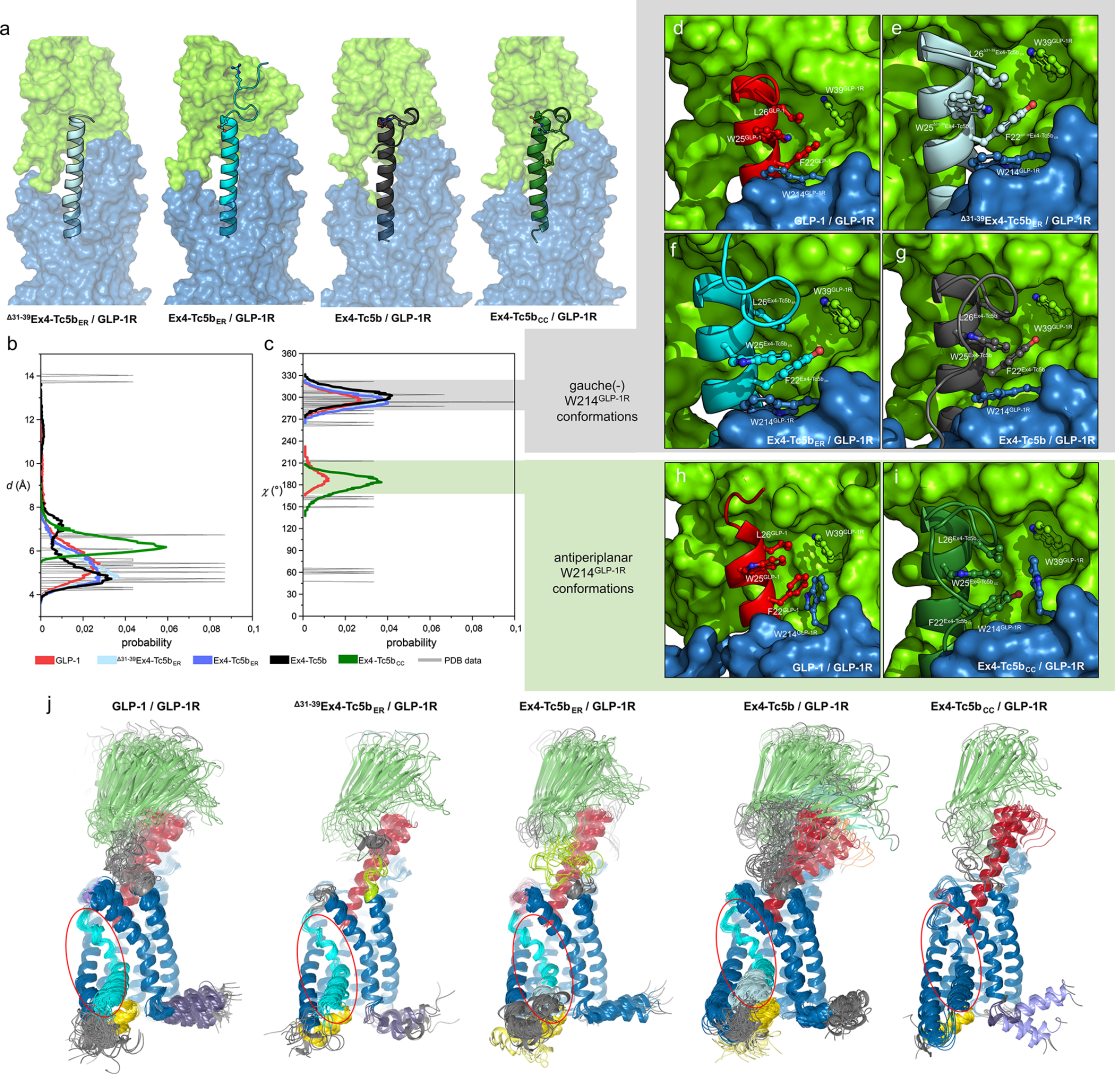
Molecular dynamics ensembles of the Ex4-related polypeptide ligands bound to GLP-1R. Molecular dynamics simulations were started from a model built from the cryo-EM structure of the human GLP-1/GLP-1R complex (PDB code 6X18) (for further details, see the Experimental Section). (a) Structural representation of Ex4-Tc5b variants with a tightening Tc fold. (b) Distance distribution of the centroids of residues W25 (ligand side) and W214^GLP-1R^ (of the ECL1 loop of the GLP-1R receptor) and (c) distribution of the χ_1_ dihedral angle of W214^GLP-1R^ in the simulated complexes and in experimentally determined structures of the human GLP-1R complexes (see data availability). Most abundant (d–g) gauche(−) orientation of W214^GLP-1R^ corresponding to an arrangement where W214^GLP-1R^ (green) and W39^GLP-1R^ (blue) of the receptor enclose the F/Y-X-X-W-L motif of the ligand and (h,i) antiperiplanar orientation of W214^GLP-1R^ in which the two receptor Trp-s are positioned near each other, leaving the ligand core exposed. (j) RBS analysis of the trajectories. Independently moving segments are colored differently. Gray coloring indicates regions that were not resolved into any of the rigid segments and, thus, move completely freely. The number of structures shown for each complex corresponds to the number of clusters needed to represent at least 95% of the snapshots, reflecting the structural heterogeneity of the systems. The TM6 segments of the ligand variants, whose dynamic properties most significantly influence signal transduction, are highlighted by a red circle.

The tightening of the Tc fold with the appearance of the salt bridge and the disulfide linker between the C-terminus and the α-helical segment is evident for the receptor-bound complexes (Figure 7a). We observed a significant decrease in the conformational heterogeneity of the ligands in the following order: Ex4-Tc5b_ER_/GLP-1R > Ex4-Tc5b/GLP-1R > Ex4-Tc5b_CC_/GLP-1R. (Table S2) In the case of the Ex4-Tc5b_ER_ /GLP-1R complex, the Tc motif adopts different open conformations, while in the case of the Ex4-Tc5b/GLP-1R and Ex4-Tc5b_CC_/GLP-1R complexes, a stable H-bond is formed between the indole NH atom of W25 and the C=O of R35, which is generally connected to the formation of a proper Tc fold.^29^ In the latter complex, an additional interaction between W25 and P36 is formed and is present in more than 30% of the snapshots. In the Ex4-Tc5b_ER_ /GLP-1R complex, we found very few instances (1.6%) along the equilibrated trajectory where the E28-R35 salt bridge/H-bond is present. However, for the Ex4-Tc5b/GLP-1R and Ex4-Tc5b_CC_/GLP-1R complexes, the D28-R35 interaction becomes prominent (present in 77.6 and 88.2% of the snapshots, respectively) (Table S3), with more than one H-bond formed between them in the majority of cases (57.4 and 60.8%, respectively) (Figure 7a).

The presence of the Trp-cage causes only modest disruption to the overall structure of the receptor, suggesting that accommodation of the ligands, even those carrying a tightly locked Tc motif, is feasible. However, a subtle conformational rearrangement is required to facilitate a perfect fit, as the Tc motif and the ECL1 loop would otherwise collide. W214^GLP-1R^ of ECL1 was previously found to be most sensitive to the presence or absence^50^ and to the exact nature of the coordinated ligands.^51−53^ In the majority of experimentally determined structures of agonist-bound complexes of human GLP-1R, W214^GLP-1R^ forms stabilizing contacts with the central W25 of Tc of the ligand. In the simulation of the GLP-1/GLP-1R complex, the W25^GLP-1^–W214^GLP-1R^ distance (measured between their centroids) samples the range of 3.7–12.4 Å with a wide maximum centered at 5.4 Å (accounting for ∼60% of the snapshots), which is in accordance with most typical distances found in the experimental structures (Figure 7b). The median distance of the W25^ligand^–W214^GLP-1R^ indole rings was found to be 5.3 Å in the ^Δ31–39^Ex4-Tc5b_ER_ and Ex4-Tc5b_ER_ receptor complexes with the distribution becoming sharper. However, in the case of Ex4-Tc5b, a cluster of snapshots with significantly longer W25^ligand^–W214^GLP-1R^ distances was found, shifting the above average to 5.7 Å and finally to 6.4 Å in the Ex4-Tc5b_CC_/GLP-1R system. In the latter complex, the Tc fold of the ligand essentially displaces residue W214^GLP-1R^ of the receptor (Figure 7b), a change also reflected in the values of the N–Cα–Cβ–Cγ (χ_1_) dihedral angle of W214 (Figure 7c).

The experimentally determined structures of various GLP-1R-complexed W214^GLP-1R^ samples both the gauche(−) (Figure 7d–g) and antiperiplanar conformations (Figure 7h,i), but the majority of the structures adhere to a gauche(−) orientation of the χ_1_ dihedral angle. The gauche(−) χ_1_ value of W214^GLP-1R^ corresponds to the W39^GLP-1R^-(F22-X-X-W25-L26)^GLP-1^-W214^GLP-1R^ sandwich topology (Figure 7d–g), with W39^GLP-1R^ and W214^GLP-1R^ enclosing the hydrophobic core of the ligand, while flipping W214^GLP-1R^ to the antiperiplanar conformation brings the two receptor indole rings close together, generating a W39^GLP-1R^-W214^GLP-1R^-(F22-X-X-W25-L26)_GLP-1_ arrangement (Figure 7h,i). In the presence of ^Δ31–39^Ex4-Tc5b_ER_, Ex4-Tc5b_ER_, and Ex4-Tc5b ligands (with a Y22-X-X-W25-L26 core), the gauche relative orientation can be found in more than 98% of the snapshots, while in the case of Ex4-Tc5b_CC_, in 97.5% of the snapshots, the χ_1_ dihedral angle of W214^GLP-1R^ is antiperiplanar. However, this does not make the W25 solvent accessible in the case of the latter because P37 of the polyproline stretch aligns parallel to the indole ring and takes the place of the displaced W214^GLP-1R^ residue of the receptor. In fact, it could be argued that the caging of the W25 (along with F/Y22 and L26) of the ligand by the receptor is part of the ligand recognition process. Two strategically placed tryptophans, W214^GLP-1R^ of the ECL1 loop and W39^GLP-1R^ of the ECD, sequester this motif in the majority of the experimentally determined structures as well as in the simulated ensembles. In the case of the Tc-carrying variants, an intraligand caging alternative is introduced. However, if the Tc is not locked, the binding mode shows only moderate changes.

### Rigid-Body Segmentation Reveals that Rigidified Tc Reduces the Dynamics of TM6, Leading to a Loss of Signaling Capacity

The stiffening and less than ideal fit of the Tc at the extracellular site also produce differences in the activation region buried in the binding pocket of TM helices. The N-terminal segment of the ligand (residues 1–14), which is immersed in the cradle formed by the TM helices of the receptor, is gradually dislodged from the position that it occupies in the GLP-1/GLP-1R complex as the Trp-cage tightens. The backbone RMSD of this segment (compared to its positions within the simulated ensemble of the GLP-1/GLP-1R complex) increases from 2.0 Å in the ^Δ31–39^Ex4-Tc5b_ER_/GLP-1R complex to 2.2 Å with the appearance of the cage in the Ex4-Tc5b_ER_ complex, to 2.4 Å with the optimized salt bridge of Ex4-Tc5b /GLP-1R, and finally to 2.8 Å in the case of Ex4-Tc5b_CC_/GLP-1R, where the disulfide linker is also present. The movement causes a subtle rearrangement of the interaction network surrounding these residues (Table S3), which is manifested in the altered dynamics of the receptor.

We recently introduced a new tool for the analysis of MD trajectories, the rigid-body segmentation (RBS) method. RBS identifies segments of the simulated systems that move in a synchronized manner, decomposing the structure into quasi-rigid parts that fluctuate with respect to each other in such a way that the sum of these independent motions reproduces the fluctuations seen in the trajectory.^54,55^ Applying RBS to the simulated trajectory of the GLP-1/GLP-1R complex, we found, as expected, that the dynamics of the ECD and TM domains are uncoupled but also that both TM6 and ECL3 fluctuate independently of the main body of the TM region (and each other), while the rest of the TMD moves in unison (Figure 7j). TM6 is a key member of the main signal transducing device of GLP-1R,^16^ so its ability to rearrange separately from the bulk of the TM region is critical for receptor function. The tightening of the Trp-cage can also be followed in the RBS results. In the case of the Ex4-Tc5b_ER_ /GLP-1R complex, the polyproline arm of the ligand moves completely freely, while in the Ex4-Tc5b/GLP-1R complex, it becomes a correlated segment (residues 33–37), although still fluctuating independently of the main helix (residues 1–30), but in the case of the Ex4-Tc5b_CC_/GLP-1R, the movement of the polyproline-II segment is bound to the rest of the ligand, forming a single RBS segment along the entire sequence (residues 1–39). This stiffening of the disulfide-bound Tc motif and its previously described dislocation within the TM region of the receptor cause a significant change in the dynamics of the complex: the motion of TM6 becomes connected to that of the rest of the TM region, losing its independent, free motion. We predict that this would severely impair signaling capacity, consistent with our finding that this variant is by far the least potent agonist of GLP-1R (Figure 7j).

## Discussion and Conclusions

A common concept in lead development is that by iteratively reducing the conformational flexibility of a promising candidate, a stronger receptor-binding potential may be achieved. Structure optimization, however, has to leave the bioactive 3D structure intact while also staying as far away as possible from pathways that lead to aggregation and/or amyloid formation. In line with this strategy, decorating hormone mimetics with optimized Tc segments that were previously shown to stabilize both the helical receptor-binding region and restrict the fluctuation of the polyproline tail is a reasonable approach. Our results, however, highlight that limiting the conformational freedom of class-B GPCR polypeptide ligands may be a misleading strategy for future developments.

The folding/unfolding of a polypeptide can be characterized effectively by spectroscopy-derived, “low-resolution” biophysical measures, which track changes but may not capture all aspects of structural transitions at the molecular level. Here, we showed that the Achilles’ heel of the Tc fold is its 3_10_ helix, the first structural motif to lose its ordered nature with increasing temperature yielding an intermediate state^56^ on the unfolding pathway. We characterized and compared at the atomic level the interaction networks of several key residues (Figure 4) that contribute to maintaining the proper 3_10_ helix geometry, thereby gatekeeping the compactness of the Tc motif. We found that optimized residue–residue interactions can delay the initialization of global unfolding by up to 20 °C. However, once the 3_10_ helix melts, subsequent unfolding steps lead to the opening and collapse of the Tc fold. At the same time, the disulfide-constrained Ex4-Tc5b_CC_ exhibits toxin-like properties by maintaining the proper Tc fold even at 90 °C, inhibiting the displacement of the polyproline helix. This stabilized fold is desirable for an extended shelf life and easier handling without refrigeration in the daily subcutaneous administration of such peptide-based medicines.

Aggregation propensity is an intrinsic and functionally critical property of human proglucagon derivative polypeptides, rendering their pharmaceutical optimization even more complex, especially because their aggregation core overlaps with their receptor-binding motif.^40^ We recently provided evidence that Ex4 of *Heloderma suspectum* exhibits extraordinary stability against aggregation when compared to polypeptides of human origin, attributable chiefly to the APR-shielding effect of Tc, which is not present in any of the human gastrointestinal hormones. To illustrate this point, here, we showed that amyloid fibrils readily form from Tc-truncated ^Δ31–39^Ex4, which thus behaves similarly to GLP-1, providing additional evidence for its shared evolutionary origin with human class-B GPCR ligands.^45^ This suggests that during the divergent evolution of lizards from the other vertebrae species, the aggregation potential of Ex4 was blocked concurrent with its function change (from hormone to toxin). At the same time, the presence of the polyproline helix is merely an aggregation-hindering factor rather than an inhibiting element. If the amyloidogenicity of the APR surpasses the aggregation-hindering effect of the polyproline tail, then amyloid formation may occur, as demonstrated by the example of Ex4-Tc5b. Here, we also found that tightly locking the Tc with dual salt and disulfide bridges (Ex4-Tc5b_CC_) resulted in the complete abolishment of aggregation potential. From the perspective of pharmaceutical development, the incorporation of a Tc motif may offer advantages for several helical and aggregation-prone GPCR-related homologue peptides, such as GLP-1, GLP-2, glucagon, VIP, and secretin. Our results confirm that the folded nature, thermostability, and amyloid resistance are increased by the presence of a well-formed Tc, and this modification could also potentially enhance the metabolic stability as was recently shown in the case of α-helical antimicrobial peptides.^30^

However, the insulin secretion assays demonstrated that the compactness and rigidity of the Tc also impact the bioactivity of exenatide derivatives. It was previously shown that a N-terminally truncated GLP-1/Ex4 derivative, which contains a disulfide bridge positioned similarly to our Ex4-Tc5b_CC_, binds in the canonical mode to the isolated ECD of the GLP-1R.^37^ However, in the presence of the entire receptor, disulfide-constrained Tc would collide with the ECL1 loop. Consequently, in the case of the experimentally determined structures of full-length GLP-1R in complex with Tc-reinforced ligands, the polyproline segments are generally absent from the final models (Figure S5) implying their liberation from the Tc fold by the binding event, and thus far, no structure containing the complex of complete GLP-1R and a covalently locked Trp-cage variant has been deposited. When comparing backbone torsion angles of different receptor-bound and free (folded and unfolded) Tc derivatives, this flexibility is shown to originate from the conformational adaptivity of the 3_10_ helix, the distribution of torsion angles shifting significantly for residue pairs −^29^GG^30^– and −^33^SG^34^– upon both binding and unfolding as compared to the folded state (Figure S6). Therefore, what was found to be the Achilles’ heel for the Tc also makes effective bioactivity possible. Our MD simulations reveal that the different levels of Tc compactness are conspicuous even within the receptor-bound complexes, causing a disturbance in the W25^ligand^–W214^GLP-1R^ interaction, rearranging the ECL1 loop (Figure 7d–i). It was recently shown that the dynamics of the TM domain reacts sensitively both to the composition of the N-terminal segment of peptide ligands through a network of transiently appearing ligand–receptor interactions, and it is also coupled to G protein activation.^23^ Here, we show that subtle displacement of identical N-terminal segments prompted by the increasingly uneasy fit of the C-terminal Tc motif of the peptides and the ECL1 loop of the receptor is sufficient to produce a similar effect. Rearrangements at the extracellular crevice of the receptor shift the N-terminal segments of the ligand within the TMD, which in turn impairs the dynamic crosstalk between the extra- and intracellular side by freezing the independent movement of TM6, thus limiting the signal transduction capacity of the receptor (Figure 7j).

Nature has chosen to create an intricate balance in the case of incretin hormones, focusing on two very different functions, requiring completely different conformations, on the same segment (residues 20–27). These few residues can create a vehicle for the receptor recognition motif when nudged into an α-helical conformation by neutral pH and the presence of the receptor and also function as the aggregation core when unfolding is initiated by the low pH of secretory vesicles, allowing tight packaging for storing. Thus, it is not wholly surprising that designing an extension such as a Tc cage that simultaneously attempts to optimize stability and preserve bioactivity of hormone mimetics requires an architecture reinforced by a strong but reversible staple (in our case the D28-R35 salt bridge) and a well-folded turn region to orient the polyproline tail (here the 3_10_ helix) that is also easily coerced into opening. In other words, design should mimic not only the composition and structure but also the dynamics and pliability of the physiological variant to create functional therapeutics.

## Experimental Section

### Bacterial Expression of Exenatide Derivatives

^Δ1–14^Ex4-Tc5b and Ex4-Tc5b variants were produced by bacterial expression. The starting cDNA was a pET-32b vector (for Ex-4) or a 3' SacII cleavage site modified pTKK19-pUBK vector^100^ construct, into which the ubiquitin (8.5 kDa) was incorporated as a fusion protein together with a polyhistidine tag (His-tag). *E. coli*, spread on an agar plate, was cultured overnight at 37 °C with shaking at 160 rpm in 50 mL of an LB medium. Expression was initiated from this preculture. An 8 mL/L cell suspension in an LB medium containing 100 μg/mL ampicillin was grown at 37 °C until an OD_600_ value of 1.2 was reached. Fusion protein production was then induced by the addition of 1 mM IPTG. After shaking for 4 h at 37 °C and 200 rpm, the cells were harvested, and the fusion proteins were purified by affinity chromatography (Profinity Ni-IMAC). The fusion protein was eluted from the column with imidazole solution, and the imidazole was removed by dialysis. The ubiquitin-linked peptides were cleaved from the fusion protein complex using yeast ubiquitin hydrolase (YUH). In a subsequent round of affinity chromatography purification, the peptides ended up in the nonbinding fraction, while the His-tagged ubiquitin remained on the column.

### Solid-Phase Peptide Synthesis

^Δ30–39^Ex4-Tc5b variants, Ex4, and GLP-1 were synthesized using our in-house developed flow chemistry-based solid-phase peptide synthesizer using the Fmoc/*t*-Bu strategy.^57,58^ A preloaded TentaGel resin containing the first C-terminal amino acid served as the starting material. Coupling reactions were carried out with OxymaPure and DIC reagents in DMF as the solvent, conducted at 80 °C under a pressure of 7–9 MPa. The oligopeptides were cleaved from the resin using a mixture of 0.25 g of phenol, 60 μL of triisopropylsilane, 125 μL of ethane-1,2-dithiol, 250 μL of water, 250 μL of thioanisole, and 5 mL of trifluoroacetic acid (TFA) at room temperature with continuous stirring for 4.5 h. TFA was then removed using a rotary vacuum evaporator, and the oligopeptides were precipitated in cold diethyl ether. After sedimentation, the ether was decanted, and the sediment was washed again with fresh ether. This purification cycle was repeated three times followed by vacuum drying.

### Purification and Analytics

The raw products of expression and peptide synthesis underwent additional processing. The peptides were dissolved in a 5:95 v/v% ACN:H_2_O mixture and filtered through a PTFE membrane with a pore size of 45 μm. The dissolved peptides were then purified using reverse-phase HPLC using a C12 column (Jasco LC-2000Plus HPLC system equipped with a Jupiter 10 μm Proteo 90 Å LC column, 250 × 10 mm) and a gradient elution (ACN/water with 0.1% TFA). All peptide compounds' analytical purity was verified by both MS (HR MS-Orbitrap) and analytical HPLC (Aeris 3.6 μm PEPTIDE XB-C18 LC column, 250 × 4.6 mm). Samples that passed analytical testing with >95% purity were combined, frozen, and lyophilized for subsequent use. For the analytical characterization of the peptide compounds, see Figure S7.

### Circular Dichroism Experiments

Circular dichroism (CD) experiments were performed using JASCO J-810 and J-1500 spectropolarimeters. JASCO Spectra Manager v1.17 and v2.14 were applied for data acquisition and processing. The cell temperature was regulated by using a Peltier-type heating system. Melting curves were recorded in the temperature range of 5–85 °C in 5 °C increments. Amyloid formation experiments were performed at a constant temperature of 37 °C. Between two data acquisition points, the amyloid samples were incubated at 37 °C and shaken at 500 rpm. All measurements were performed in 1.0 mm path length quartz cuvettes. For determining melting curves, the sample concentrations and pH were set at 20–30 μM and pH = 7.0. In amyloid aggregation experiments, samples were prepared at concentrations of 200–300 μM with 150 mM NaCl at pH values of 4.5 and 7.4. Each spectrum was the average of three scans collected at a spectral scan rate of 50 nm/min, a bandwidth of 1 nm, and a step resolution of 0.2 nm over the wavelength range of 185–260 nm (far-UV). Spectra used to monitor amyloid formation were truncated at 195 nm due to an increased signal-to-noise ratio resulting from an increased ionic strength. All spectra were corrected by subtracting the solvent spectrum and smoothed using the Savitzky–Golay method with a convolution width of seven. The raw ellipticity data (mdeg) were converted into mean residue molar ellipticity units ([θ]_MR_/deg cm^2^ dmol^–1^). Exact concentrations were determined by using a Nanodrop Lite UV–vis spectrophotometer at 280 nm. To determine the folded fraction (*F*%), the temperature-dependent spectrum collection of 65 CD curves was analyzed using Convex Constraint Analysis Plus software.^59^ Software is available online: https://www.chem.elte.hu/departments/jimre/.

### Nuclear Magnetic Resonance Experiments

The NMR samples were prepared according to the same protocol. Aqueous solutions of lyophilized proteins were prepared at concentrations of 0.5–0.8 mM, typically in a total volume of 400–800 μL, containing 8–10% D_2_O, 1% NaN_3_, and DSS as an internal reference alongside. The pH of the samples was adjusted to 7.0 using 0.1 M NaOH and HCl, and the samples were transferred to 5 mm-diameter normal or Shigemi tubes. NMR measurements were performed on a 16.4T (700 MHz) Bruker Avance III spectrometer, equipped with either a *z*-gradient 5 mm inverse TCL probe or a Prodigy TCI H&F-C/N-D probe. During each temperature-dependent measurement series, samples of each peptide variant were measured under identical conditions at all five investigated temperatures: 277 K, 4 °C; 288 K, 15 °C; 299 K, 26 °C; 310 K, 37 °C; 321 K, 48 °C. At each measurement point, a 1D ^1^H spectrum was first recorded to confirm the uniformity of the sample. Then, after recording of the 2D ^1^H–^1^H homonuclear spectra (which usually took one or two days), another 1D ^1^H spectrum was obtained as a control. A comparison of the two ^1^H spectra was used to determine whether any degradation had occurred during the acquisition, but none was observed. Full proton resonance assignments and cross peak analyses were carried out using water-suppressed 2D ^1^H–^1^H homonuclear DQF-COSY (cosygpprqf), TOCSY (mlevgpph19), and NOESY (noesygpph19) standard Bruker experiments. The resolutions were set to 2048 × 512, with 32 or 64 scans. For TOCSY measurements, a spinlock of d9 = 80 ms, and for NOESY measurements, a mixing time of d8 = 150 ms was applied. Spectrum processing (phase adjustment, baseline correction, and reference calibration) was performed using Topspin software versions 3.5–4.0.7, and spectrum assignment was performed using CCPNMR software version 2.4.1.^60^

### Structure Characterization-Based Secondary Chemical Shifts

The chemical shifts of the ^1^H nuclei are indicative of the surrounding molecular environment, and thus, the extent of secondary structure formation can be estimated by analyzing the secondary chemical shifts (SCS) of selected nuclei within the Tc fold. Secondary chemical shifts were determined using the following equation: SCS = δ_obs_ – δ_rc_, where δ_obs_ is the measured chemical shift and δ_rc_ is the random coil reference. To determine the random coil chemical shifts of the Hα protons, the Poulsen predictor^61^ was used, which complexly comprises several literature methods.^62−64^ The SCS_helix_ values are the sum of the absolute values of individual Hα proton SCS from helical segments ranging from residues 2 to 13. The aromatic ring current of Trp induces an anisotropic magnetic field that causes the chemical shifts of the nuclei above and below the indole plane to drift toward smaller values, while those within the plane shift toward higher values. To determine the compactness and degree of folding of the Tc motifs, the protons^29,31^ most affected by the aromatic ring current, W25Nε1, L21Hα, G30Hα2, P31Hβ2, R35Hα, P37Hα, P37Hβ2, P38Hδ1, P38Hδ2, and W25Nε1, were chosen to calculate SCS_TC_ values (the residue positions are according to the Ex4 numbering). The random coil reference chemical shifts for these protons were obtained from the literature.^65^

### Normalization of the Tc-Fold Descriptors

To facilitate the comparison of melting curves obtained through different techniques, the measures ([Θ_222nm_]_MR_, folded fraction %, SCS_helix_, and SCS_TC_) describing the structural components of the Tc fold were standardized as follows: , where *X* represents the measured biophysical value of a given 25-residue-long Tc variant (TC) at a specific temperature (°C).

### Structure Determination by NOESY-Derived ^1^H–^1^H Distance Restraints

CCPNMR 2.4.1 was used to generate a list of distance restraints from assigned NOESY cross peaks. The ^1^H–^1^H distances were estimated using the software’s standard protocol, which derives restraint distances from the volume of assigned NOESY cross peaks using the distance function of volume intensity^–1/6^. The software calculates a reference distance (3.2 Å) from the average of the volume intensities and then estimates the restraint distances based on this reference ratio. The lower and upper distance limits were set at 1.72 and 8.00 Å, respectively. The restraint lists were then applied to distance restraint-based structure calculations, using ARIA 2.3.1 software.^66^ The resulting data output was then imported back into CCPNMR for further evaluation and refinement. The standard ARIA simulated annealing protocol was followed, and after 8 consecutive iterations, during which after each iteration, the software retained the top 7 structures out of 20 based on the total energy criterion, a water refinement of the final structure ensemble was performed. The all-atom RMSD along the sequences and the dihedral angles used to plot the Ramachandran plots were determined using the built-in functions of CCPNMR. PyMOL 2.3.2 was used for visualization of the structure ensembles.

### Amyloid Aggregation Propensity Monitored by Thioflavin-T (ThT) Experiments

The exact peptide content percentage of the lyophilized peptides was determined by using a Nanodrop Lite UV–vis spectrophotometer at 280 nm. The exact amount of peptides was then dissolved in water, and the pH was adjusted to either pH 4.2–4.5 or pH 7.2–7.4, with 0.1 M NaOH. The samples were then filtered using a 0.44 μm filter-equipped Eppendorf tube and centrifuged for 2 min at 13,000 rpm to ensure that only dissolved monomers remained. Next, 160 μL of the peptide solution was added to black-walled 96-well microplates with flat bottoms (Greiner Bio-One, Frickenhausen, Germany). ThT (Acros Organics, Thermo Fisher, Geel, Belgium) stock solutions (50 μM) were prepared by adjusting the pH of distilled water to pH 7 and 4 and then filtering through a 0.44 μm filter. The exact concentration was determined by UV spectrometry using a Jasco V-660 spectrophotometer (Tokyo, Japan) at 412 nm with an extinction coefficient of 31.600 M^–1^ cm^–1^. Prior to ThT kinetic measurements, 20 μL of ThT stock solution and 20 μL of 1500 μM NaCl solution were added to the wells, resulting in final peptide concentrations of 1 or 5 mg mL^–1^. The plate was sealed to prevent evaporation. A SpectraMax iD3 microplate reader (Molecular Devices, Sunnyvale, CA, USA) was used to control the experimental conditions (37 °C, orbital shaking at medium intensity) and to collect fluorescence data over 3 days. The excitation and emission wavelengths were set at 445 and 490 nm, respectively. The emitted fluorescence was measured from the bottom of the microplate with medium photomultiplier tube sensitivity (PMT) settings and an integration time of 400 ms. All measurements were conducted in triplicate. The average fluorescence intensity of the peptide-free ThT sample was used as a background and subtracted from the average fluorescence intensity of the peptide-containing samples at each measurement point. The mean and standard deviation of ThT fluorescence intensities were plotted as a function of time using Origin software version 2022b and Excel software version 2020.

### Atomic Force Microscopy Measurements

Ten μL aliquots were taken from the ThT and CD measurement samples at the end point of the experiments, after 3 days and 2 weeks of agitation, respectively. These aliquots were then spread on freshly cleaved mica surfaces and dried overnight in a vacuum desiccator. In some cases, the spread samples were washed with pH-adjusted water to remove any salts that might crystallize during the drying process and potentially complicate the AFM micrograph recording. The surface morphology was analyzed using a FlexAFM microscope system (Nanosurf AG, Liestal, Switzerland) operating in dynamic mode, controlled by Nanosurf control software C3000 version 3.10.4. Micrographs were taken using Tap150GD-G cantilevers (BudgetSensors Ltd., Sofia, Bulgaria) with a nominal tip radius of less than 10 nm. Prescreening was run before data collection to avoid significant height variations in the surface topology. Data collection was performed at different locations within each sample. Images were recorded with a maximum window size of 10 × 10 μm at a resolution of 512 pixels/line. Gwyddion 2.62 software was used to process the AFM data and generate the images.

### Insulin Secretion Enhancing Activity Assay

Rat β-cell lines of INS-1E cells were preserved in liquid nitrogen. Before experiments, the cells were cultured according to a standard protocol^67^ in a humidified atmosphere containing 5% CO_2_ at 37 °C in a medium composed of RPMI 1640 supplemented with 10% heat-inactivated fetal bovine serum, 1 mM sodium pyruvate, 2 mM glutamine, 10 mM HEPES, 50 μM 2-mercaptoethanol, 100 units mL^–1^ penicillin, 100 μg mL^–1^ streptomycin, and 250 ng mL^–1^ amphotericin-B. The cell culture was grown at 37 °C with constant medium exchange until 80% confluence of the culture flask was reached and then passaged. Experiments were initiated after the fifth passage followed by cell counting in a Bruker chamber using Trypan blue staining. The total cell count was determined using the following formula: *N* = *n* × dilution × total volume of the cell suspension × 10,^4^ where *n* is the average of two independent cell counts. The cell suspension (1–1 mL) was passaged onto a 24-well plate, and then, the cells were maintained with continuous medium exchange until 80% confluence was reached in each well. The cells were first incubated in a glucose-free RPMI 1640 medium incubator for 2 h and washed twice with a Krebs–Ringer bicarbonate HEPES buffer (KRBH), and then, 2.5 mM glucose was added after 30 min. After another 30 min of incubation, the pipetted and centrifuged supernatant was used as a reference for low-glucose cell response. After another KRBH wash, following half an hour, 15 mM glucose and then 20 nM of the protein to be tested were pipetted onto the cells, and the samples were incubated for half an hour. The concentration of insulin in the centrifuged supernatant was determined by the enzyme-linked immunosorbent assay (ELISA) method according to the protocol of Abcam’s Human Insulin ELISA kit (ab100578, Abcam, Cambridge, UK). The amount of secreted insulin was determined from the absorbance values measured at 450nm using the calibration curve provided by the kit. To achieve the appropriate absorbance range, the supernatant was diluted 20 times. Simultaneously, a total of 14 absorbance measurements were evaluated from 2-2 passed cell cultures per peptide.

### Molecular Dynamic Simulations

Molecular dynamics (MD) simulations were started from a common model built from the cryo-EM structure of the human GLP-1/GLP-1R complex^16^ (PDB code 6X18). This model contained the entire GLP-1R receptor plus a single helix (α5) of the G_α_s subunit of the coupled G protein to preserve the orientation of the TM helices. This approach proved effective as the conformation of the TMD region remained stable during the MD simulations, resulting in ∼1 Å RMSD for the backbones of all TM helices (Table S2). The ECD domains also remained quite similar to those measured for the GLP-1/GLP-1R complex but fluctuated rather freely with respect to the membrane-embedded TM helices increasing the overall RMSD. Similar flexibility of the ECD domain can be seen in the collection of the experimentally determined structures the ligand-bound complexes of GLP-1R (Table S2). To account for this conformational heterogeneity, a dynamic reference state, that of the equilibrium cluster of the GLP-1/GLP-1R complex, was used, so that the different conformational ensembles derived using the Ex4-Tc5b variants were compared with the conformational ensemble obtained by using the physiological ligand. The models of the Trp-cage-containing ligands were built from the crystal structure of the ECD-bound exenatide^18^ (PDB code 3C5T) and the NMR structure of its solution-phase,^25^ standalone form (PDB code 1JRJ), with the necessary side chain mutations introduced manually. Missing segments were built and optimized by Monte Carlo multiple minimum searches, as well as the membrane model (a system containing 129 POPC lipid molecules) using the Schrödinger Suite (Schrödinger Release 2019-3: Maestro, Schrödinger, LLC, New York, NY, 2019). Simulations were carried out as implemented in GROMACS^68^ using a custom version of the AMBER-ff99SBildnp* force field^69^ for the protein components and the lipid force field for describing the POPC membrane molecules.^70^ The system was solvated with TIP3P water molecules in triclinic boxes (78.4 × 77.6 × 136.5 Å), removing all solvent molecules from the model that were placed within 5.5 Å of the TM helices (residues 146–168, 179–197, 228–246, 268–283, 313–328, 352–370, and 390–405, located by CCTOP^71^) or the hydrophobic core of POPC molecules. The total charge was neutralized, and the physiological salt concentration (0.15 M) was set using Na^+^ and Cl^–^ ions. Energy minimization of the starting structures was followed by sequential relaxation of the constraints on the protein atoms in three steps while restraining the P atoms of the POPC molecules and an additional NVT step allowing all atoms free movement (all typically 20 ns long, using a time step of 2 fs). Simulations were carried out at 310 K and 1 bar. Two copies of 500 ns NPT trajectories were collected for the ^Δ31–39^Ex4-Tc5b_ER_/GLP-1R, Ex4-Tc5b_ER_/GLP-1R, Ex4-Tc5b/GLP-1R, and Ex4-Tc5b_CC_/GLP-1R systems and 4 copies for the reference GLP-1/GLP-1R complex for further analysis. Analysis of the interaction networks of the MD-derived model systems revealed that the sequence differences between GLP-1 and Ex4-Tc5b lead to a more intense interaction between the TM domain-immersed N-terminal segment of the ligand and the stalk region of the receptor (the segment connecting the ECD and the TM domains) in the complexes containing Ex4-Tc5b variants due to the transient Q13-S136^GLP-1R^, E16-R131^GLP-1R^/R134^GLP-1R^, Q24-G^132GLP-1R^, and K27-E127^GLP-1R^ associations, which are absent in the GLP-1/GLP-1R complex.

### Rigid-Body Segmentation

For the rigid-body segmentation^54,55^ of the MD trajectories, a 2.5 Å distance threshold (cluster radius or “clr” argument) and a neighbor count of 5 (cluster neighbors or “cln” argument) were used in the DBSCAN clustering algorithm. Segmentation was based on clustering of the Cα–Cα distance standard deviation matrix as a precomputed metric. Frames were analyzed in 100 ps intervals over the equilibrium trajectory.

## Data Availability

The processed CD, ThT, and ELISA data collected in this study are provided in the source data file. The chemical shift list, the restraints list, and the calculated structure ensembles are deposited in Biological Magnetic Resonance Data Bank (BMRB) and the RCSB Protein Data Bank (PDB) under the following accession codes: ^Δ1–14^Ex4-Tc5b: 34932/9G22 (277K), 34933/9G2N (288K), 34934/9G2O (299K), 34935/9G31 (310K), and 34936/9G32 (321K); ^Δ1–14^Ex4-Tc5b_ER_: 34937/9G5P (277K), 34927/9G0M (288K), 34928/9G0N (299K), 34930/9G20 (310K), and 34931/9G21 (321K); ^Δ1–14^Ex4-Tc5b_CC_: 34941/9GDL (277K), 34943/9GDN (288K), 34944/9GDT (299K), 34945/9GDU (310K), and 34949/9GE1 (321K); ^Δ1–14^Ex4-Tc5b_QR_: 34950/9GE9 (277K), 34951/9GEB (288K), 34952/9GEC (299K), and 52579 (310K). Analysis of experimental data sets for Figure 7 was carried out based on PDB structures 5NX2, 6B3J, 6ORV, 6LN2, 6VBC, 6X18, 6X19, 6X1A, 6X0X, 7C2E, 7EVM, 7LCJ, 7LCK, 7LCI, 7LLL, 7DUQ, 7DUR, 7E14, 7FIM, 7KI0, 7KI1, 7RGP, 7S1M, 7VBH, 7VBI, 7X8R, 7X8S, 8JIP, 8JIR, and 8JIS. The code used here for rigid-body segmentation analysis is available at the link https://github.com/fazekaszs/rigid_body_segmentation.

## Supporting Information Available

(Figures S1–S7) Temperature-dependent far-UV CD curves, melting curves of truncated Tc variants, Ramachandran plots, aggregation propensity at neutral pH, structures of ligand–receptor complexes, Comparison of the dihedral angles, and analytical characterization of the applied polypeptides; (Tables S1–S3) 3_10_ helix geometry defining NOE cross peaks, backbone RMSD along the equilibrated MD trajectories, and H-bond formation in the various MD simulated systems (PDF)
Processed CD, ThT, and ELISA data collected in this study (source data file) (XLSX)
Protein components of the cluster midstructures of the MD simulations (ZIP)

## Abbreviations

ACN: acetonitrile
AFM: atomic force microscopy
APR: aggregation-prone region
CD: circular dichroism spectroscopy
cDNA: circular DNA
COSY: correlation spectroscopy
cryo-EM: cryogenic electron microscopy
DIC: *N*,*N*′-diisopropylcarbodiimide
DMF: dimethylformamide
DQF-COSY: double quantum filtered COSY
DSS: sodium 4,4-dimethyl-4-silapentane-1-sulfonate
*E. coli*: *Escherichia coli*
ECD: extracellular domain
ECL: extracellular loop
ELISA: enzyme-linked immunosorbent assay
Ex4: exendin-4, exenatide
*F*%: folded fraction
Fmoc: 9-fluorenylmethoxycarbonyl
GLP-1: glucagon-like peptide-1
GLP-1R: GLP-1 receptor
GPCR: G protein-coupled receptor
HEPES: 4-(2-hydroxyethyl)-1-piperazineethanesulfonic acid
HPLC: high-pressure liquid chromatography
ICL: intracellular loop
IMAC: immobilized metal affinity chromatography
IPTG: isopropyl β-d-1-thiogalactopyranoside
KRBH: Krebs–Ringer bicarbonate HEPES
MD: molecular dynamics
MS: mass spectrometry
MR: nuclear magnetic resonance spectroscopy
NOE: nuclear Overhauser effect
NOESY: nuclear Overhauser effect spectroscopy
OD: optical density
POPC: 1-palmitoyl-2-oleoyl-*sn*-glycero-3-phosphocholine
PTFE: polytetrafluoroethylene
RBS: rigid-body segmentation
RMSD: root-mean-square deviation
rpm: revolutions per minute
SCS: secondary chemical shift
T2DM: type 2 diabetes mellitus
*t*-Bu: *tert*-butyl
Tc: tryptophan-cage
TFA: trifluoroacetic acid
ThT: thioflavin-T
TM: transmembrane helix
TMD: TM domain
TOCSY: total correlation spectroscopy
VIP: vasoactive intestinal peptide
YUH: yeast ubiquitin hydrolase
[θ]_MR_: mean residue molar ellipticity
