# Supplementary material for: Influence of Trp-Cage on the Function and Stability of GLP-1R Agonist Exenatide Derivatives
Source: J Med Chem. 2024 Sep 10;67(18):16757–72. doi: 10.1021/acs.jmedchem.4c01553 (PMC11440607; doi:10.1021/acs.jmedchem.4c01553)
Supplement: Supplementary file 1 — jm4c01553_si_001.pdf [file jm4c01553_si_001.pdf]

Supporting Information

The Influence of Trp-Cage on the Function and Stability of GLP-1R Agonist Exenatide Derivatives

Dániel Horváth[2,3], Pál Stráner[2,3], Nóra Taricska[2,3], Zsolt Fazekas[3,4], Dóra K. Menyhárd[1,2,3*], András Perczel[1,2,3]*

[1] Medicinal Chemistry Research Group, HUN-REN Research Centre for Natural Sciences, Magyar Tudósok Körútja 2, H-1117 Budapest, Hungary
[2] HUN-REN–ELTE Protein Modeling Research Group, ELTE Eötvös Loránd University, Pázmány Péter sétány 1/A, H-1117 Budapest, Hungary.
[3] Laboratory of Structural Chemistry and Biology, ELTE Eötvös Loránd University, Pázmány Péter sétány 1/A, H-1117 Budapest, Hungary
[4] Hevesy György PhD School of Chemistry, ELTE Eötvös Loránd University, Pázmány Péter sétány 1/A, H-1117 Budapest, Hungary.

Email: perczel.andras@ttk.elte.hu; karancsine.menyhard.dora@ttk.hu

Table of contents:

Figure S1: Temperature dependent far-UV CD curves S2
Figure S2: Melting curves of truncated Tc variants S2
Figure S3: Ramachandran plots generated from the calculated average structures S3
Figure S4: Aggregation propensity at neutral pH S4
Figure S5: Structures of ligand-receptor complexes of Exenatide derivatives S5
Figure S6: Comparison of the dihedral angles (Trp25-Pro38) of different Trp-cage conformations S6-S7
Figure S7: Analatycal characterization of the applied polypeptides S7-S9
Table S1: 3_{10}-helix geometry defining NOE cross peaks at 4 and 27°C S10
Table S2: Backbone RMSD along the equilibrated MD trajectories S11
Table S3: H-bond formation in the various MD simulated systems S11

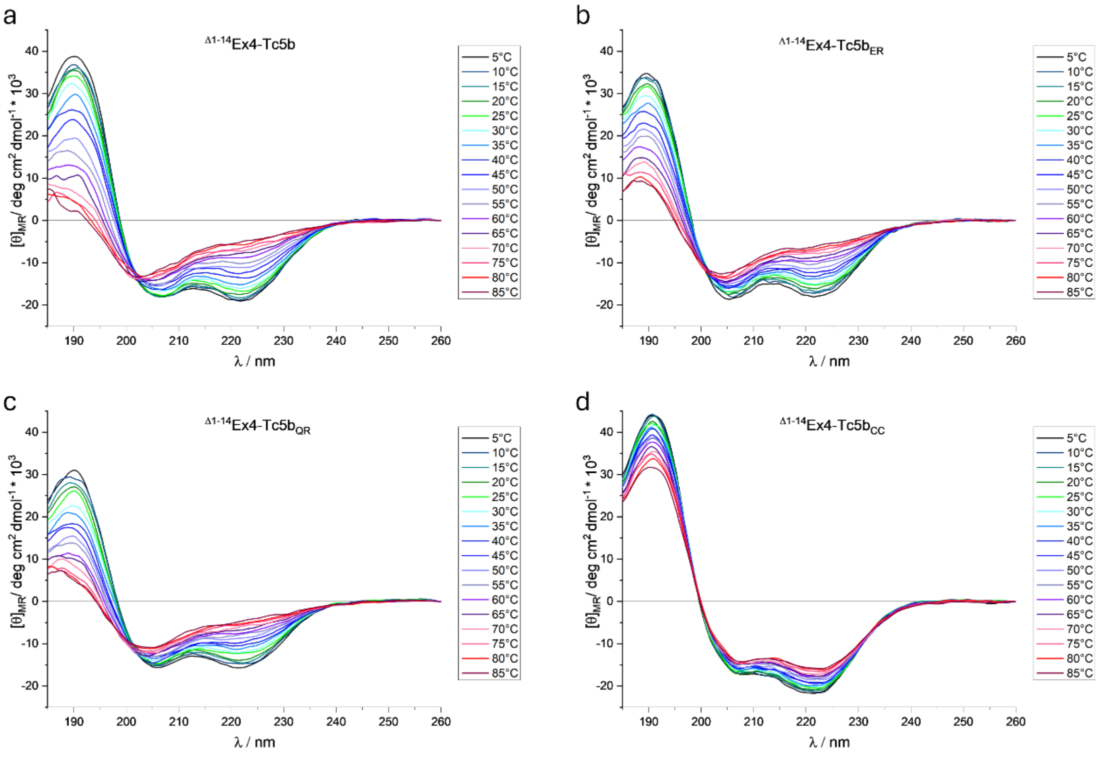

Figure S1: Temperature dependent far-UV CD curve ensembles of **(a)** $^{\Delta 1\text{-}14}$Ex4-Tc5b, **(b)** $^{\Delta 1\text{-}14}$Ex4-Tc5b$_{ER}$, **(c)** $^{\Delta 1\text{-}14}$Ex4-Tc5b$_{QR}$, **(d)** $^{\Delta 1\text{-}14}$Ex4-Tc5b$_{CC}$.

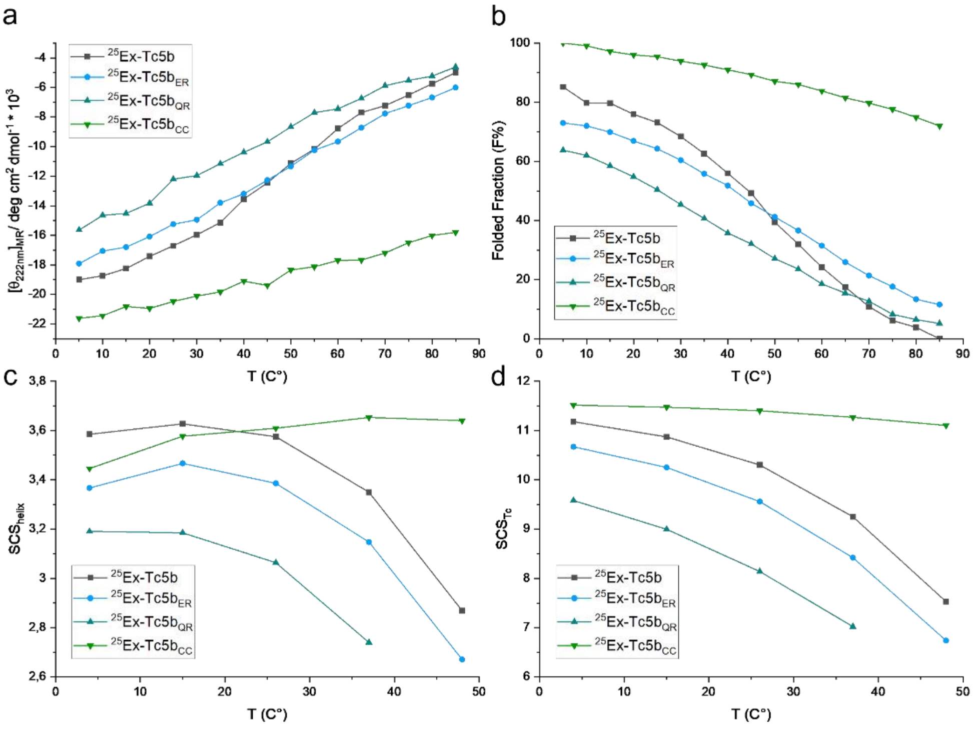

Figure S2: Comparison of measured melting curves by ECD (a,b) and NMR (c,d) datasets. **(a)** Molar ellipticity values at 222nm $[\Theta_{222nm}]_{MR}$ and **(b)** folded fraction values (F%) derived from deconvolution are obtained through ECD spectroscopy. Sums of secondary chemical shift values describing helicity (SCS_{helix}) **(c)** and Tc-fold compactness (SCS_{TC}) **(d)** are determined by NMR measurements. Note that the NMR-derived datasets cover only the temperature range up to 48°C, compared to 85°C for the ECD.

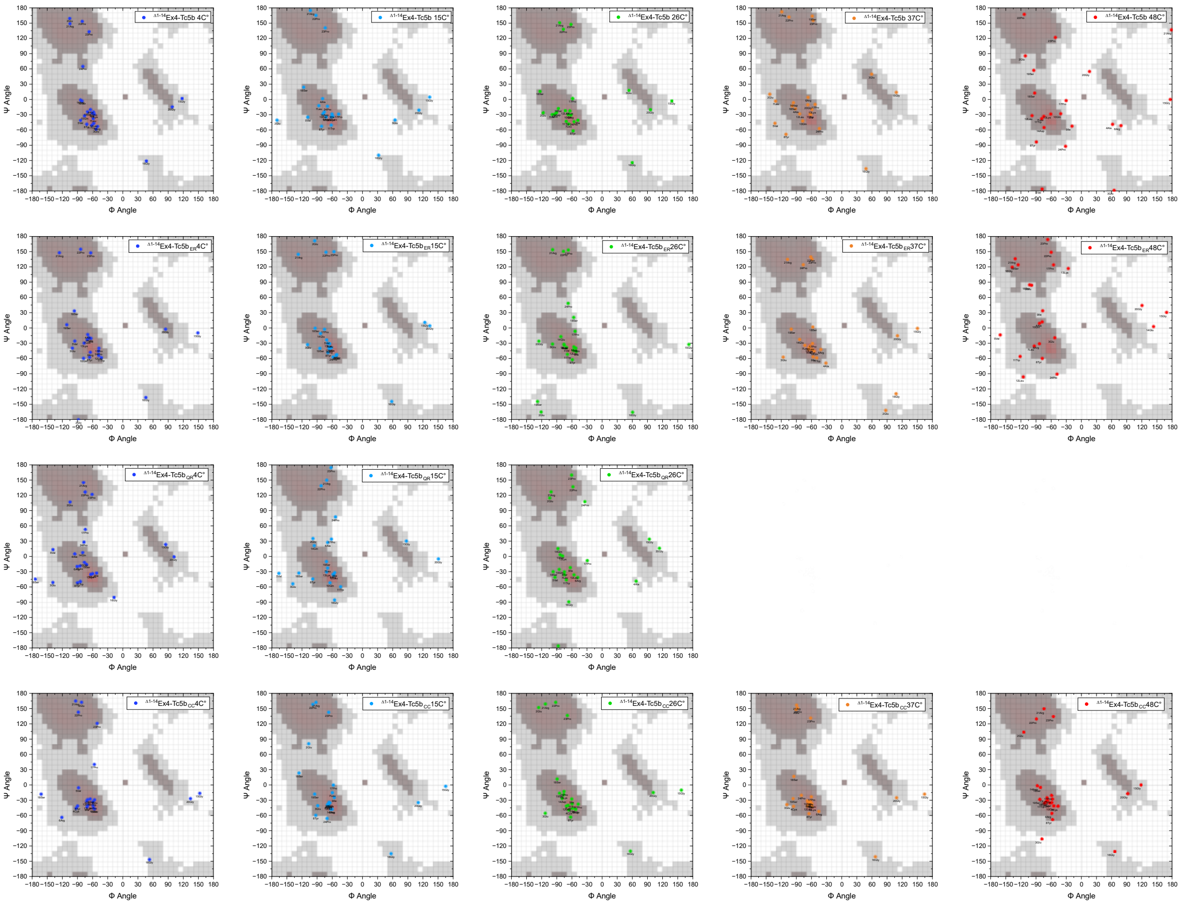

Figure S3: Ramachandran plots generated from the calculated average structures of Ex-Tc5b variants. First row: $^{\Delta1\text{-}14}$Ex4-Tc5b, second row: $^{\Delta1\text{-}14}$Ex4-Tc5b$_{ER}$, third row: $^{\Delta1\text{-}14}$Ex4-Tc5b$_{QR}$, fourth row: $^{\Delta1\text{-}14}$Ex4-Tc5b$_{CC}$. Backbone dihedrals are color-coded as a function of temperature from left to right as 4C° - dark blue, 15C° - light blue, 26C° - green, 37C° - orange, 48C° - red. The brown and grey areas represent the favored and allowed regions, respectively. Dihedrals of the peptide backbones are more frequently located outside of the favored regions when the calculated structure is more ambiguous, often due to the lack of structure-determining NOE restraints. The vast majority of backbone coordinates typically fall within the region's characteristic of α-helix and 3_{10} helix conformations. Dihedrals of prolines are typically found within the region corresponding to polyproline helices, while glycine and the generally unstructured Glu2 at the N-terminus are dispersed throughout the plot.

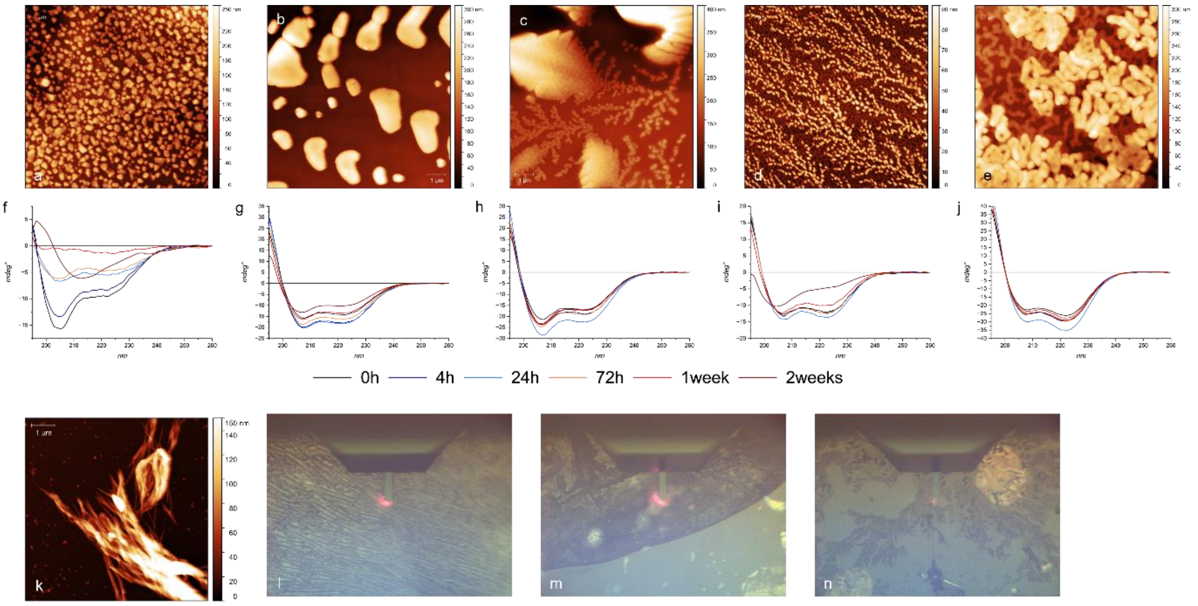

Figure S4: The recorded AFM micrographs were taken of the end states of the samples **(a)** GLP-1, **(b)** $^{\Delta 31\text{-}39}$Ex4, **(c)** Ex4 **(d)** Ex4-Tc5b **(e)** Ex4-Tc5b$_{CC}$) used for ThT measurements, with a concentration of 1 mg ml^{-1}, at neutral pH after 72 hours of orbital shaking at 37 C°. Time-resolved CD spectroscopy monitors the changes in the secondary structure of **(f)** GLP-1, **(g)** $^{\Delta 31\text{-}39}$Ex4, **(h)** Ex4 **(i)** Ex4-Tc5b **(j)** Ex4-Tc5b$_{CC}$ at a concentration of 1 mg ml^{-1}, neutral pH, 37 C° over a period of two weeks. **(k)** The amyloid fibrils observed in the end-state of the CD sample GLP-1 after 2 weeks of continuous stirring with a magnetic bar exhibit a more lumpy and tangled morphology compared to the samples acquired from the acidic ThT end-states. This difference in morphology is likely due to the fact that the latter samples were agitated under milder conditions (orbital shaking) and for a shorter period of time. Examples of intense salt-crystal formation on the mica surface of vacuum-dried samples are shown in images captured by the built-in AFM camera, both without **(l,m)** and with **(n)** washing after the transfer of the sample to mica.

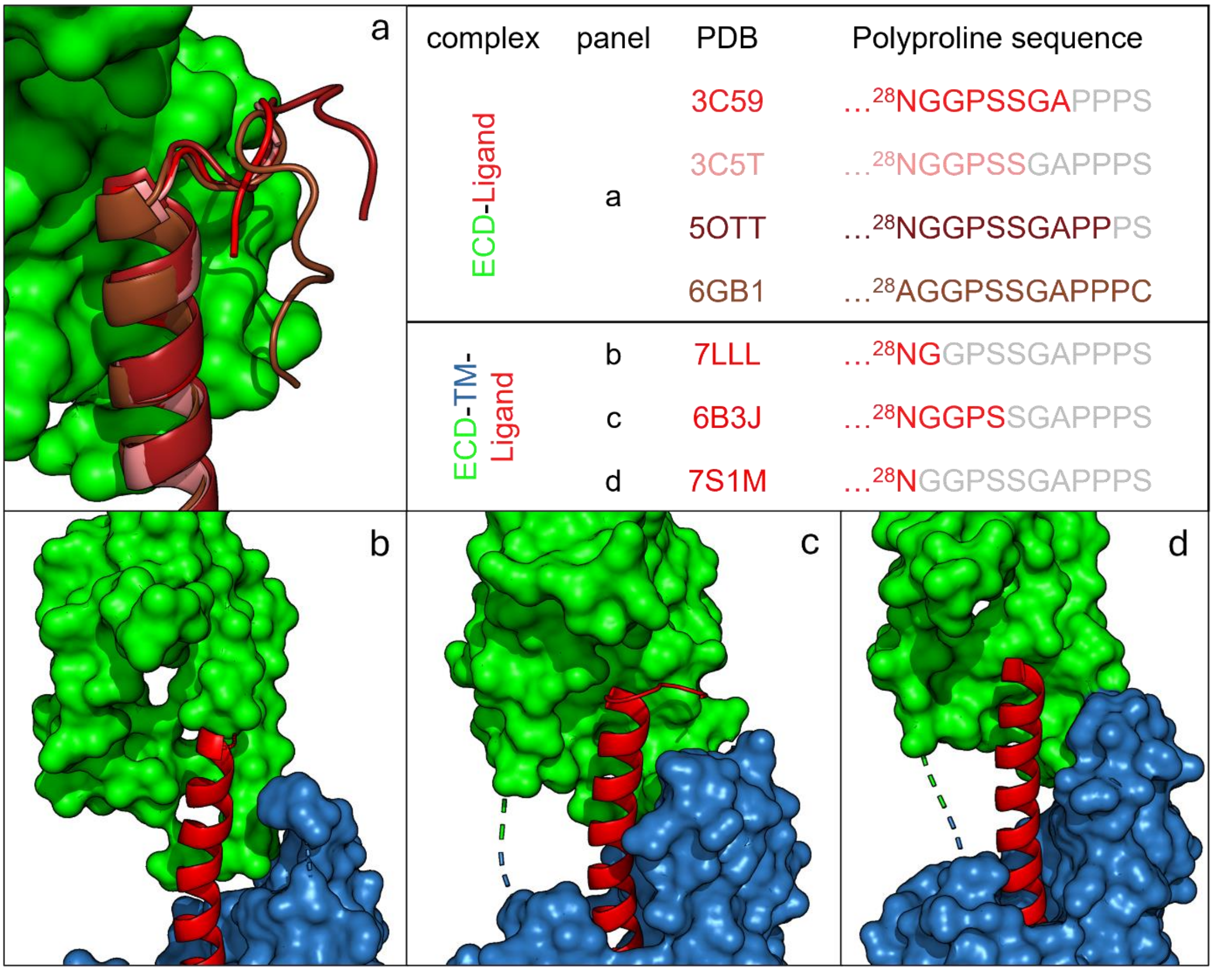

complex	panel	PDB	Polyproline sequence
ECD-Ligand	a	3C59	…^{28}NGGPSSGAPPPS
		3C5T	…^{28}NGGPSSGAPPPS
		5OTT	…^{28}NGGPSSGAPPPS
		6GB1	…^{28}AGGPSSGAPPPC
ECD-TM-Ligand	b	7LLL	…^{28}NGGPSSGAPPPS
	c	6B3J	…^{28}NGGPSSGAPPPS
	d	7S1M	…^{28}NGGPSSGAPPPS

Figure S5: Crystal or Cryo-EM structures of ligand-receptor complexes of Exenatide derivatives with either **(a)** the single extracellular domain (ECD) or **(b-d)** the full GLP-1R are found in the PDB database. Grey shading in the sequences highlights regions with missing electron-density maps, indicating high flexibility in those areas.

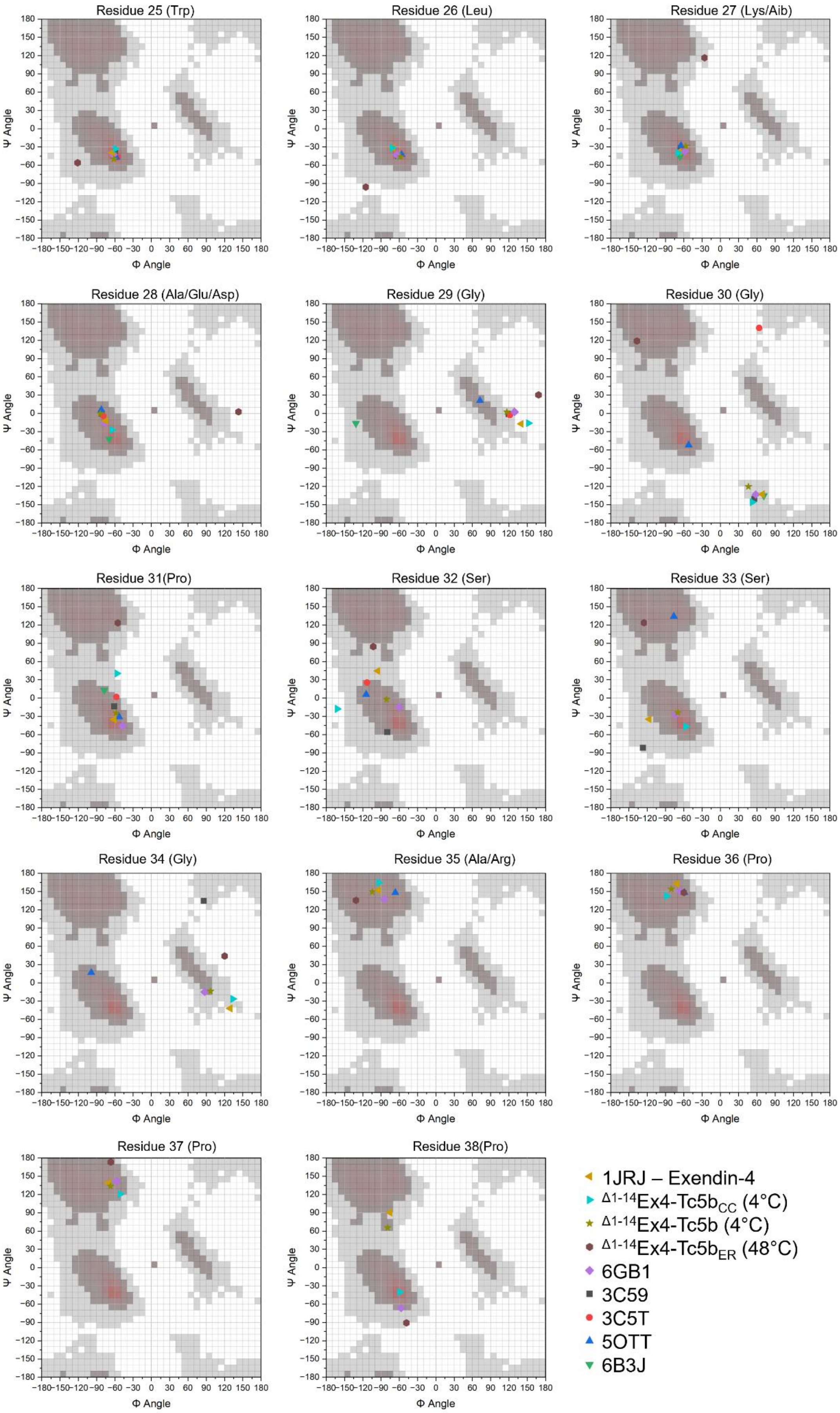
Residue 25 (Trp)
Residue 26 (Leu)
Residue 27 (Lys/Aib)
Residue 28 (Ala/Glu/Asp)
Residue 29 (Gly)
Residue 30 (Gly)
Residue 31(Pro)
Residue 32 (Ser)
Residue 33 (Ser)
Residue 34 (Gly)
Residue 35 (Ala/Arg)
Residue 36 (Pro)
Residue 37 (Pro)
Residue 38(Pro)
Ψ Angle
Φ Angle
1JRJ – Exendin-4
Δ1-14Ex4-Tc5bCC (4°C)
Δ1-14Ex4-Tc5b (4°C)
Δ1-14Ex4-Tc5bER (48°C)
6GB1
3C59
3C5T
5OTT
6B3J

Figure S6: A residue-by-residue comparison of the dihedral angles (Trp25-Pro38) of different Trp-cage conformations belonging to free and GLP-1R-bound ligand states. To describe the dihedral distribution of the Trp-cages in the free-state, folded ligands, we selected 1JRJ (Exendin-4), $^{\Delta1\text{-}14}$Ex4-Tc5b$_{CC}$, and $^{\Delta1\text{-}14}$Ex4-Tc5b (the latter two at 4°C); for the free unfolded-state, we applied $^{\Delta1\text{-}14}$Ex4-Tc5b$_{ER}$ at 48°C. The structural models 6GB1, 3C59, 3C5T, and 5OTT represent ligands bound to the ECD, while in 6B3J, the full receptor is involved. In the receptor-bound models, several C-terminal proximal residues are missing due to the structural ambiguity of these models (as highlighted in Figure S5). According to the plots between residue 25-28 (the C-proximal end of the α-helix) and residue 35-38 (the poly-proline helix), the dihedral distributions are low, indicating that the ligands adopt identical structures regardless of whether they are bound to the receptor or not. The only exception is $^{\Delta1\text{-}14}$Ex4-Tc5b$_{ER}$ at 48°C, which exhibits divergent dihedrals at the helical segment, indicating the collapse of the α-helix. Interestingly, its poly-proline helix distribution does not differ from the folded states, suggesting that the polyproline is stiff enough to avoid unfolding at 48°C. Between residue 29 (Gly) and residue 34 (Gly), the dihedrals of the 3_{10}-helix exhibit the largest divergence between the folded, free, and the receptor-bound states, highlighting the structural rearrangement that occurs upon ECD binding in this region.

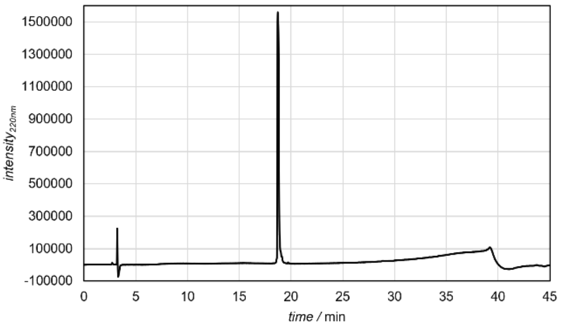

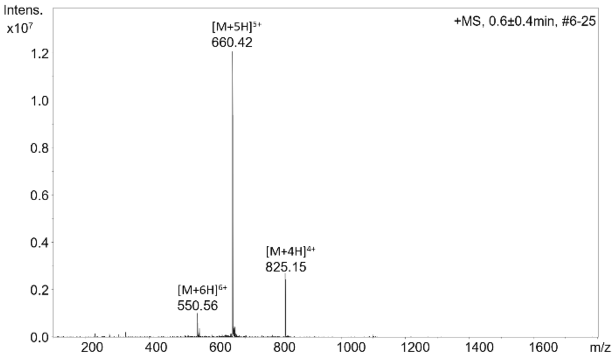

peptide	t_R(min)	Area	Height	Area%	Height%	Calculated m/z	Measured m/z
GLP-1	18.720	15293912	1550952	95.382	95.301	3297.64	3297.10

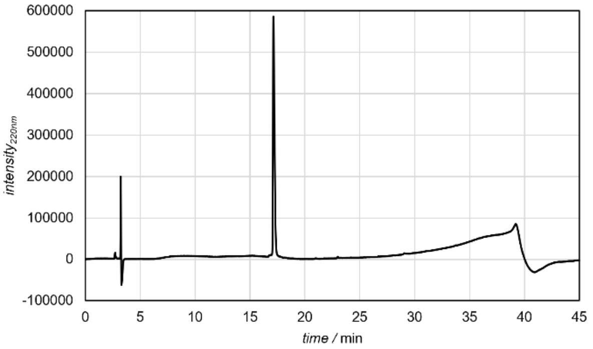

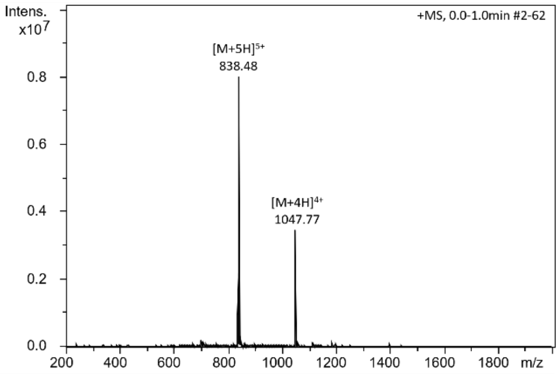

peptide	t_R(min)	Area	Height	Area%	Height%	Calculated m/z	Measured m/z
Exendin-4	17.133	5619414	579759	98.914	99.145	4187.56	4187.40

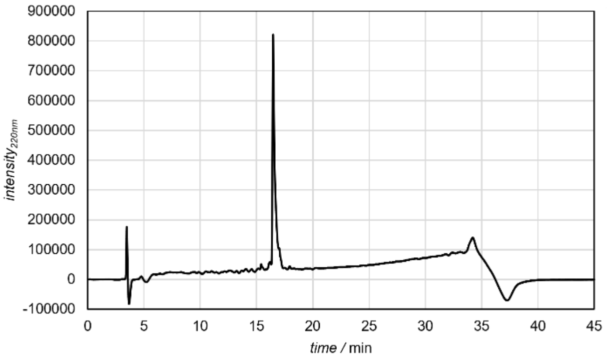

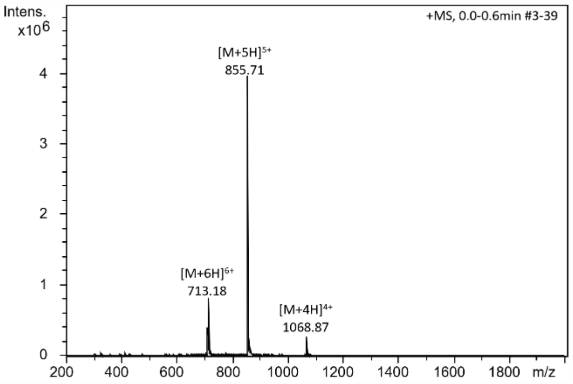

peptide	t_R(min)	Area	Height	Area%	Height%	Calculated m/z	Measured m/z
Ex4$_{DR}$	16.472	11934423	786346	97.072	97.086	4273.66	4273.08

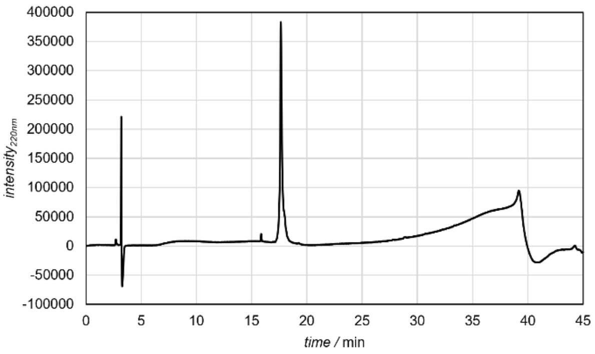

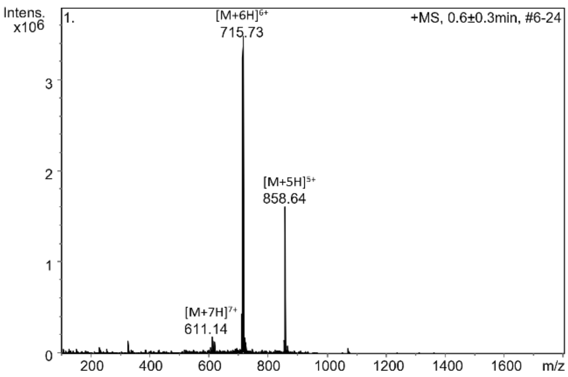

peptide	t_R(min)	Area	Height	Area%	Height%	Calculated m/z	Measured m/z
Ex4-Tc5b	17.640	5315694	378046	98.884	96.671	4288.67	4288.38

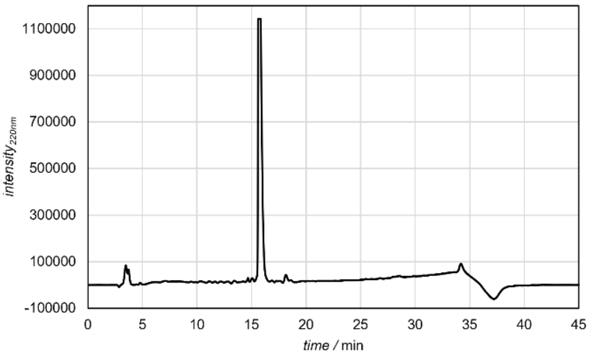

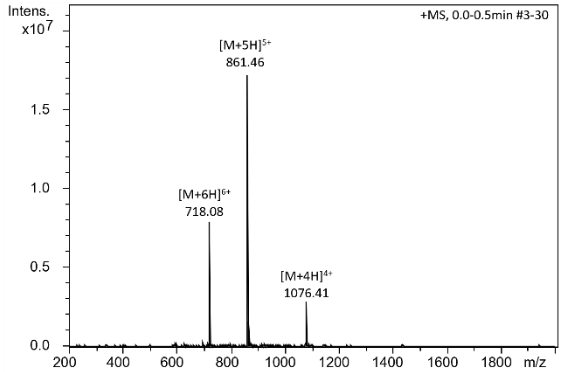

peptide	t_R(min)	Area	Height	Area%	Height%	Calculated m/z	Measured m/z
Ex4-Tc5b$_{ER}$	15.830	27966437	1129712	95.599	95.870	4302.70	4302.30

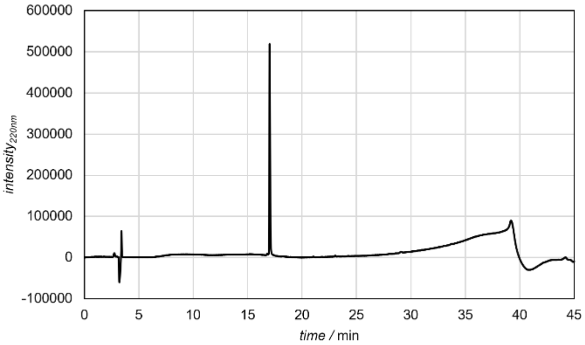

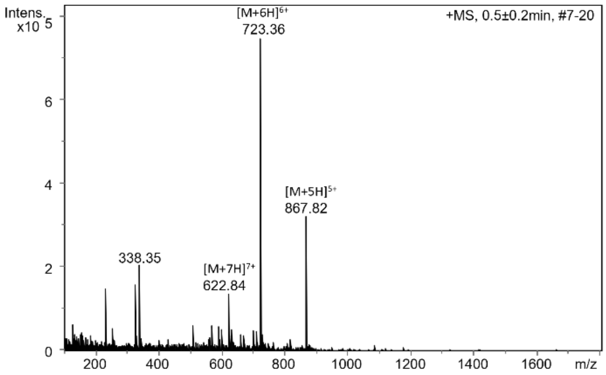

peptide	t_R(min)	Area	Height	Area%	Height%	Calculated m/z	Measured m/z
Ex4-Tc5b$_{CC}$	17.013	2500402	511034	98.963	99.285	4334.79	4334.16

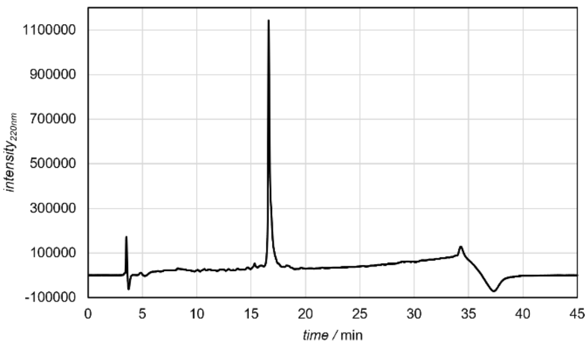

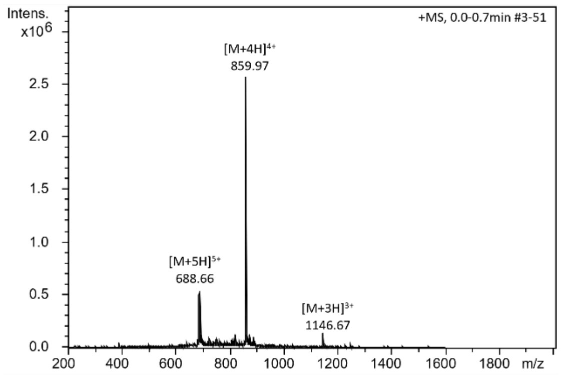

peptide	t_R(min)	Area	Height	Area%	Height%	Calculated m/z	Measured m/z
$^{\Delta 31\text{-}39}$Ex4-Tc5b$_{ER}$	16.613	15281957	1103568	98.872	98.690	3438.79	3438.30

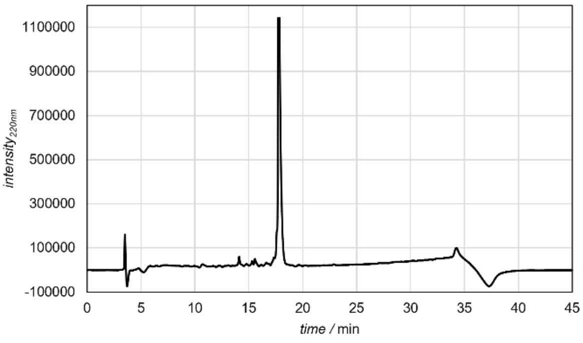

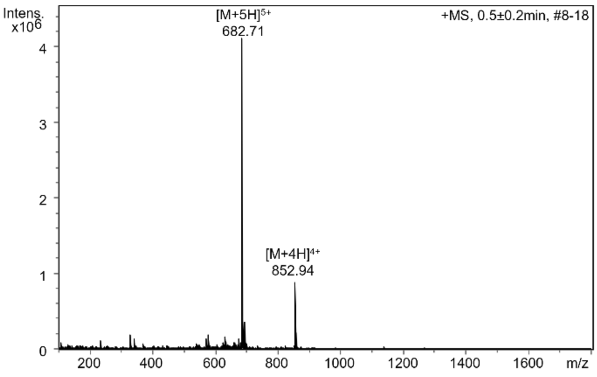

peptide	t_R(min)	Area	Height	Area%	Height%	Calculated m/z	Measured m/z
$^{\Delta 31\text{-}39}$Ex4	17.782	21878469	1118116	95.641	96.636	3408.76	3408.55

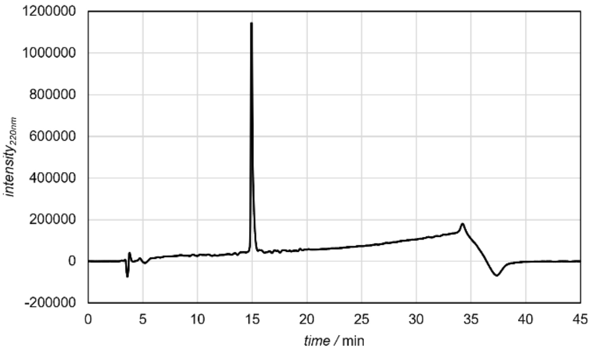

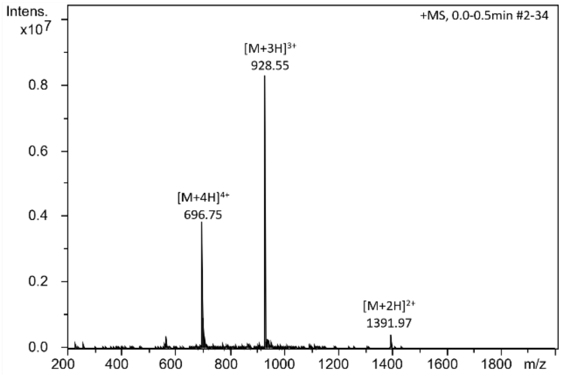

peptide	t_R(min)	Area	Height	Area%	Height%	Calculated m/z	Measured m/z
$^{\Delta 1\text{-}14}$Ex4-Tc5b$_{ER}$	14.943	14966281	1105546	95.458	98.043	2783.06	2782.65

Figure S7: Analytical characterization of applied polypeptides by HPLC and mass spectrometry. For further details see the Experimental section. The m/z value of 338.35 in MS spectra of Ex4-Tc5b$_{CC}$ is a result of general contamination of the local mass spectrometer and does not relate to the polypeptide.

Table S1: The observed NOE cross peaks assigned to the proton resonance pairs in the table define the 3_{10}-helix geometry. Cross peaks highlighted in black were observed at 4°C, those in red were observed at both 4°C and 27°C, and those in purple were observed only at 27°C.

$^{\Delta 1-14}$Ex4-Tc5b$_{\mathrm{CC}}$	$^{\Delta 1-14}$Ex4-Tc5b	$^{\Delta 1-14}$Ex4-Tc5b$_{\mathrm{ER}}$	$^{\Delta 1-14}$Ex4-Tc5b$_{\mathrm{QR}}$
14 AspH - 19 SerHba	14 AspHa - 19 SerHa	14 GluHb* - 19 SerHba	14 GlnHe2a - 19 SerHb*
14 AspH - 19 SerHbb	14 AspHba - 19 SerHba	14 GluH - 11 TrpHa	14 GlnHga - 10 GlnHe2b
14 AspHba - 19 SerHa	14 AspHba - 19 SerHbb	14 GluHb* - 11 TrpHa	11 TrpHe1 - 19 SerHg
14 AspHba - 19 SerHbb	14 AspHbb - 19 SerHba	14 GluH - 10 GlnHa	19 SerHg - 21 ArgH
14 AspHbb - 19 SerHa	14 AspHbb - 19 SerHa	14 GluHb* - 10 GlnHe2a	11 TrpHd1 - 21 ArgHba
14 AspHbb - 19 SerHba	14 AspHbb - 19 SerHbb	14 GluHga - 10 GlnHe2b	11 TrpHd1 - 21 ArgHbb
14 AspHbb - 19 SerHbb	14 AspH - 11 TrpHa	14 GluHgb - 10 GlnHga	11 TrpHd1 - 21 ArgHga
14 AspHba - 19 SerHba	14 AspHba - 11 TrpHa	14 GluHgb - 10 GlnHgb	11 TrpHe1 - 21 ArgH
14 AspH - 11 TrpH	14 AspHba - 11 TrpHd1	14 GluHgb - 10 GlnHe2a	11 TrpHe1 - 21 ArgHba
14 AspH - 11 TrpHa	14 AspHbb - 11 TrpHa	14 GluHgb - 10 GlnHe2b	11 TrpHe1 - 21 ArgHbb
14 AspHba - 11 TrpHa	14 AspHbb - 11 TrpHd1	11 TrpHe1 - 19 SerHba	11 TrpHe1 - 21 ArgHga
14 AspHbb - 11 TrpHa	14 AspH - 10 GlnHe2a	21 ArgH - 19 SerH	11 TrpHe1 - 21 ArgHgb
14 AspHba - 10 GlnHe21	14 AspH - 10 GlnHe2b	21 ArgH - 19 SerHbb	11 TrpHe1 - 21 ArgHd*
14 AspHbb - 10 GlnHe21	14 AspHba - 21 ArgHh1*	11 TrpHd1 - 21 ArgHb*	11 TrpH - 10 GlnHa
14 AspHbb - 10 GlnHe22	14 AspHbb - 21 ArgHh1*	11 TrpHd1 - 21 ArgHga	11 TrpH - 10 GlnHb*
14 AspHbb - 21 ArgHe	19 SerH - 11 TrpHe1	11 TrpHd1 - 21 ArgHgb	11 TrpHbb - 10 GlnH
19 SerHba - 11 TrpHd1	19 SerHba - 11 TrpHd1	11 TrpHd1 - 21 ArgHd*	11 TrpH - 10 GlnH
19 SerHbb - 11 TrpHe1	19 SerHbb - 11 TrpHd1	11 TrpHe1 - 21 ArgH	
19 SerHg - 11 TrpHd1	19 SerHg - 11 TrpHd1	11 TrpHe1 - 21 ArgHa	
19 SerHg - 11 TrpHe1	19 SerHba - 11 TrpHe1	11 TrpHe1 - 21 ArgHb*	
19 SerHg - 11 TrpHz2	19 SerHbb - 11 TrpHe1	11 TrpHe1 - 21 ArgHgb	
19 SerHbb - 11 TrpHd1	19 SerHg - 11 TrpHe1	11 TrpHe1 - 21 ArgHga	
19 SerHba - 21 ArgH	19 SerHa - 21 ArgH	11 TrpH - 10 GlnHa	
19 SerHbb - 21 ArgH	19 SerHba - 21 ArgH	11 TrpH - 10 GlnHba	
19 SerHg - 21 ArgH	19 SerHbb - 21 ArgH	11 TrpH - 10 GlnHgb	
19 SerHa - 21 ArgHda	19 SerHg - 21 ArgH	11 TrpHbb - 10 GlnH	
19 SerHbb - 21 ArgHg*	11 TrpH - 21 ArgHh1*		
11 TrpHd1 - 21 ArgH	11 TrpHa - 21 ArgHh1*		
11 TrpHd1 - 21 ArgHba	11 TrpHbb - 21 ArgHh1*		
11 TrpHd1 - 21 ArgHbb	11 TrpHd1 - 21 ArgH		
11 TrpHd1 - 21 ArgHga	11 TrpHd1 - 21 ArgHba		
11 TrpHd1 - 21 ArgHgb	11 TrpHd1 - 21 ArgHbb		
11 TrpHd1 - 21 ArgHda	11 TrpHd1 - 21 ArgHga		
11 TrpHd1 - 21 ArgHdb	11 TrpHd1 - 21 ArgHgb		
11 TrpHd1 - 21 ArgHe	11 TrpHd1 - 21 ArgHda		
11 TrpHe1 - 21 ArgH	11 TrpHd1 - 21 ArgHdb		
11 TrpHe1 - 21 ArgHa	11 TrpHd1 - 21 ArgHe		
11 TrpHe1 - 21 ArgHba	11 TrpHd1 - 21 ArgHh1*		
11 TrpHe1 - 21 ArgHbb	11 TrpHe1 - 21 ArgH		
11 TrpHe1 - 21 ArgHga	11 TrpHe1 - 21 ArgHa		
11 TrpHe1 - 21 ArgHgb	11 TrpHe1 - 21 ArgHba		
11 TrpHh2 - 21 ArgHda	11 TrpHe1 - 21 ArgHbb		
11 TrpHh2 - 21 ArgHdb	11 TrpHe1 - 21 ArgHga		
11 TrpHh2 - 21 ArgHe	11 TrpHe1 - 21 ArgHgb		
21 ArgHga - 10 GlnHe22	11 TrpHe1 - 21 ArgHh1*		
11 TrpH - 10 GlnH	21 ArgHh1* - 10 GlnHga		
11 TrpH - 10 GlnHa	21 ArgHh1* - 10 GlnHgb		
11 TrpH - 10 GlnHba	11 TrpH - 10 GlnHa		
11 TrpH - 10 GlnHbb	11 TrpH - 10 GlnHba		
11 TrpH - 10 GlnHga	11 TrpH - 10 GlnHbb		
11 TrpH - 10 GlnHgb	11 TrpH - 10 GlnHga		
11 TrpHba - 10 GlnH	11 TrpH - 10 GlnHgb		
11 TrpH - 10 GlnHe22	11 TrpHba - 10 GlnH		
	11 TrpHbb - 10 GlnH		

Table S2: Backbone RMSD along the equilibrated MD trajectory with respect to the starting structure of the simulations (model built based on the experimentally determined structure of the GLP-1/GLP-1R complex (PDB code: 6x18)) using the folded core-structure of the ECD and TM domains: residues 29-47, 66-129 in the ECD domain, and the TM helices formed by residues 146-168, 179-197, 228-246, 268-283, 313-328, 352-370 and 390-405. Clustering was carried out for the backbone and C_β atoms of the ligand (numbers referring to the ^{30}Ex-Tc5b$_{ER}$ complex were omitted because this system does not contain a Trp-cage).

	RMSD (ECD)	RMSD (TM)	RMSD (ECD+TM)	cluster distribution*
^{30}Ex-Tc5b$_{ER}$ /GLP-1R	0.93	0.96	1.43	-
Ex-Tc5b$_{ER}$ /GLP-1R	1.70	0.95	2.29	125 / 31
Ex-Tc5b /GLP-1R	1.75	1.25	2.34	26 /5
Ex-Tc5b$_{CC}$ /GLP-1R	1.60	0.93	1.90	6 / 1

* number of clusters accounting for all the snapshots / 95% of the snapshots of the equilibrated trajectory

Table S3: H-bond formation in the various MD simulated systems. Intra-molecular contacts of W25 of the ligand are shown in yellow, the Tc stabilizing salt-bridge/H-bond in red. Ligand-receptor intermolecular interactions formed between the N-terminal segment of the ligand and specific residues of the receptor are shown in blue, while interactions between various segments of the ligand and receptor are shown in green ("Ligand" refers to the entire ligand, "GLP1-R" to the full-length receptor model).

	**GLP-1**	**$^{\Delta31\text{-}39}$Ex4-Tc5b$_{ER}$**	**Ex4-Tc5b$_{ER}$**	**Ex4-Tc5b**	**Ex4-Tc5b$_{CC}$**
frequency of H-bond formation:					
W25^{Ligand} … R35^{Ligand}	-	-	0%	81.9%	91.0%
W25^{Ligand} … P36^{Ligand}	-	-	0%	9.7%	32.4%
E28/D28^{Ligand} … R35^{Ligand}	-	-	1.6%	77.6%	88.2%
N-term^{Ligand} …Y24$^{GLP\text{-}1R}$	46.6%	48.3%	7.6%	48.6%	59.7%
N-term^{Ligand} …E364$^{GLP\text{-}1R}$	59.5%	51.0%	16.4%	51.4%	58.7%
N-term^{Ligand} …E387$^{GLP\text{-}1R}$	55.9%	98.6%	80.2%	30.5%	78.0%
H1^{Ligand} … Q234$^{GLP\text{-}1R}$	15.3%	29.3%	50.8%	3.4%	54.0%
average number of H-bonds:					
H1^{Ligand} … GLP-1R	2.0	2.6	1.7	1.4	2.6
res. 1-15 ^{Ligand} … GLP-1R	13.4	13.4	14.1	11.8	13.1
Ligand … res. 127-140 $^{GLP\text{-}1R\ stalk}$	2.3	4.2	7.5	4.3	4.3
Ligand … GLP-1R	16.5	17.7	22.2	16.5	19.7
